# A Review on Metal–Organic Framework-Derived Porous Carbon-Based Novel Microwave Absorption Materials

**DOI:** 10.1007/s40820-020-00582-3

**Published:** 2021-01-12

**Authors:** Zhiwei Zhang, Zhihao Cai, Ziyuan Wang, Yaling Peng, Lun Xia, Suping Ma, Zhanzhao Yin, Yi Huang

**Affiliations:** National Institute for Advanced Materials Tianjin Key Laboratory of Metal and Molecule Based Material Chemistry, Key Laboratory of Functional Polymer Materials, Collaborative Innovation Center of Chemical Science and Engineering (Tianjin), School of Materials Science and Engineering, Nankai University, Tianjin, 300350 People’s Republic of China

**Keywords:** Metal–organic frameworks, Porous carbon, Microwave absorption material, Reflection loss, Effective absorption bandwidth

## Abstract

The development of microwave absorption materials (MAMs) is a considerable important topic because our living space is crowed with electromagnetic wave which threatens human’s health. And MAMs are also used in radar stealth for protecting the weapons from being detected. Many nanomaterials were studied as MAMs, but not all of them have the satisfactory performance. Recently, metal–organic frameworks (MOFs) have attracted tremendous attention owing to their tunable chemical structures, diverse properties, large specific surface area and uniform pore distribution. MOF can transform to porous carbon (PC) which is decorated with metal species at appropriate pyrolysis temperature. However, the loss mechanism of pure MOF-derived PC is often relatively simple. In order to further improve the MA performance, the MOFs coupled with other loss materials are a widely studied method. In this review, we summarize the theories of MA, the progress of different MOF-derived PC‑based MAMs, tunable chemical structures incorporated with dielectric loss or magnetic loss materials. The different MA performance and mechanisms are discussed in detail. Finally, the shortcomings, challenges and perspectives of MOF-derived PC‑based MAMs are also presented. We hope this review could provide a new insight to design and fabricate MOF-derived PC-based MAMs with better fundamental understanding and practical application.

## Highlights

- The theoretical knowledge in the field of microwave absorption is summarized in detail.
- The recent progress of metal–organic frameworks-derived porous carbon-based nanocomposites as microwave absorption materials is reviewed.

## Graphical abstract

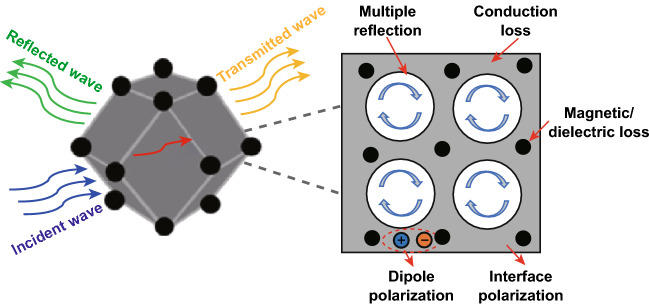

## Introduction

The rapid development of science and technology made many kinds of electronic devices become irreplaceable role in human’s daily life [1–7]. However, the electronic devices make the space rife with electromagnetic waves (EMWs) [8–14]. The EMWs become a new and more hazardous source of pollution as water, air and noise pollution [15–17]. On the one hand, the undesirable EMW may make strong interference to the nearby instruments, causing their malfunctioning and signal interruption. On the other hand, EMW may harm human’s health which cause some disease such as cancer and endocrine disorder [18, 19]. Besides, plants can be inactive, variation and even die with the strong EMW radiation [20, 21]. It is urgently need for human to solve the problem of EMW pollution, while in the field of military, many advanced weapons such as warcraft are the key target of the enemy. EMW stealth technology of military equipment is a crucial solution to evade detection and attack. Coating of MAMs on military equipment is an effective anti-detection method [22]. Therefore, the exploration of high-performance MAMs is of great significance in both civil and military fields.

Recently, MAMs have received much attention because they have the ability to attenuate EMW. They can convert EMW into thermal energy or other forms of energy to dissipate [23, 24]. The ideal MAMs are often multiple loss mechanisms and they required to have lightweight, thin thickness, wide absorption bandwidth and strong absorption characteristics [25, 26].

Metal–organic frameworks (MOFs) are a kind of crystalline porous material with periodic network structure, which is composed of inorganic metal center (metal ion or metal cluster) and organic ligand connected by self-assembly [27–30]. Due to large amounts of organic ligands that could be used, MOFs have various of compositions and structures. As we know, more than 20,000 MOFs have been reported so far [31, 32]. MOFs have attracted lots of research interest due to their performance diversity, which are potential to extensive uses in many fields, such as electrochemical energy storage [33, 34], catalysis [35, 36], purification [37, 38] and sensing [39, 40]. Moreover, with MOFs as the precursor, carbon and metal-based compounds can be generated in situ by high-temperature pyrolysis in an inert atmosphere [28, 41, 42]. Fortunately, the morphologies of MOFs are still well preserved after pyrolysis [43, 44]. The MOF-derived PC nanocomposites also possess the desirable properties from MOFs [45] such as their tunable chemical structures, large specific surface area, uniform pore distribution, diverse morphology and chemical stability, which enable MOF-derived PC to be an ideal candidate for MA.

MOF-derived PC-based nanocomposites have been widely studied in the field of MAMs, but the attenuation mechanism may be relatively simple. In order to improve the attenuation performance, they usually coupled with other lossy materials. Based on the above views, how to design and prepare MOF-derived PC-based MAMs is now a hot research topic [46, 47]. In this review, we summarize the recent progress of several MOF-derived PC-based nanocomposites as MAMs such as Co, Ni, Fe, Zn, Cu, Ti, Zr and rare-earth (RE) MOF-derived PC-based nanocomposites. Besides the pure MOF, multi-metal MOF or tunable chemical composition incorporated with other loss material had also been fabricated as MAMs. Furthermore, MAMs with different morphology had been reviewed. Finally, we put forward some personal insights into the current status and perspectives in the future research direction.

## Theories of Microwave Absorption

**Fig. 1:**
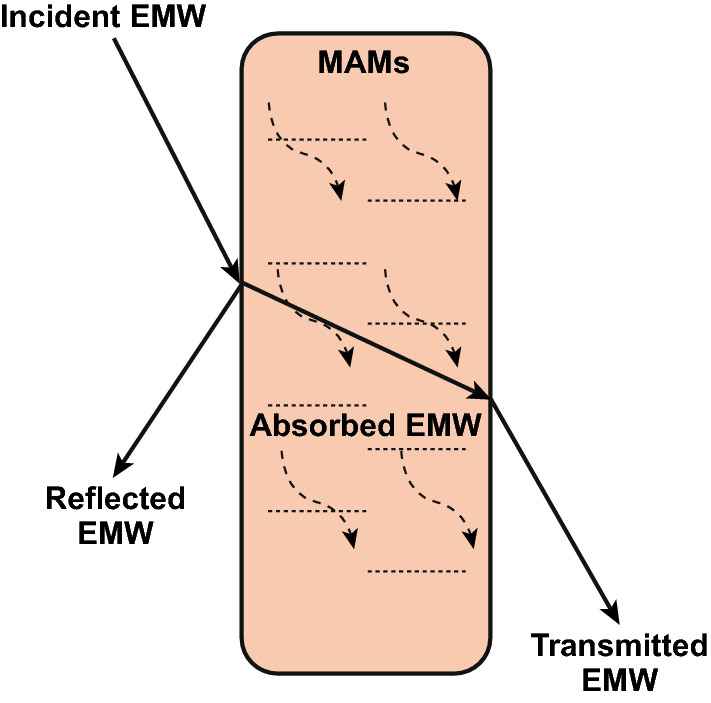
Schematic diagram of interaction between MAMs and microwaves

When the incident EMW contacts with the surface of the MAMs, as shown in Fig. 1, three situations may happen. Part of the incident EMW reflects on the surface of the MAMs (reflected EMW), part of it goes to the interior of the MAMs and absorbed by the MAMs (adsorbed EMW), and the rest of the EMW goes through the MAMs (transmitted EMW) [48]. When designing MAMs, researchers expect the incident EMW to be dissipated as much as possible inside the MAMs to reduce reflected EMW and transmitted EMW. Therefore, a good MAM usually needs to meet two conditions: good impedance matching and strong EMW attenuation ability [49, 50]. The good impedance matching requires incident EMW goes into the MAMs as much as possible and reduces the reflection on the material surface [51]. The ideal impedance matching requires that the complex permittivity (*ɛ*_*r*_= *ɛ′* − *jɛ″*) is equal to the complex permeability (*μ*_*r*_=* μ′* − *jμ″*). In the formula, *ɛ′* and *μ′* represent the ability to store electrical and magnetic energy, while *ε″* and *µ″* refer to the loss of electrical and magnetic energy [52, 53]. It is not easy to meet this requirement. We can artificially adjust electromagnetic parameters (*ɛ* and *μ*) to improve the impedance matching. The EMW attenuation ability means the loss capacity of the EMW which enters into the interior of MAMs [24, 54, 55].

Reflection loss (*RL*) is often used to evaluate the EMW absorption ability. For example, the *RL* value of − 10 dB is comparable to 90% of MA, and the *RL* value of − 20 dB is comparable to 99% of MA. The *RL* is calculated by the transmission line theory, which is shown as follows [56–60]:

1 $$Z = \left| {Z_{\text{in}} /Z_{0} \left| = \right|\sqrt {u_{r} /\varepsilon_{r} } \tanh \left[ {j\left( {2\pi fd /c\sqrt {\varepsilon_{r} u_{r} } } \right)} \right]} \right|$$

2 $$R_{L} = 20\log \left| {\left( {Z_{\text{in}} - Z_{0} } \right)/\left( {Z_{\text{in}} + Z_{0} } \right)} \right|$$

*Z*_0_ is the impedance of free space (377 Ω), *Z*_in_ is the input impedance of MAMs, *f* is the frequency of EMW, *d* is the thickness of the MAMs, and *c* is velocity of light, respectively. When *Z* = 1, the wave impedance of the MAMs is exactly the same as that of the free space. The incident EMW can enter the MAMs completely without reflected wave. Therefore, *Z *= 1 is an ideal situation. When the value of *Z* is equal or close to 1, it is beneficial for improving MA ability [61, 62]. The delta-function is another method to evaluate the EM impedance matching degree. The equation is shown as follows [63–65]:

3 $$\left| \Delta \right| = \left| {\sinh^{2} \left( {Kfd} \right) - M} \right|$$

4 $$K = \frac{{4\pi \sqrt {\mu_{r }^{\prime } \varepsilon_{r}^{\prime } } \times \sin \left( {\frac{{\delta_{e} + \delta_{m} }}{2}} \right)}}{{c \times \cos \delta_{e} \times \cos \delta_{m} }}$$

5 $$M = \frac{{4\mu_{r}^{\prime } \varepsilon_{r}^{\prime } \cos \delta_{e} \times \cos \delta_{m} \sqrt {\mu_{r}^{\prime } \varepsilon_{r}^{\prime } } \times \sin \left( {\frac{{\delta_{e} + \delta_{m} }}{2}} \right)4\mu_{r}^{\prime } \varepsilon_{r}^{\prime } \cos \delta_{e} \times \cos \delta_{m} \sqrt {\mu_{r}^{\prime } \varepsilon_{r}^{\prime } } \times \sin \left( {\frac{{\delta_{e} + \delta_{m} }}{2}} \right)}}{{\left( {\mu_{r}^{\prime } \cos \delta_{e} - \varepsilon_{r}^{\prime } \cos \delta_{m} } \right)^{2} + \left[ {\tan \left( {\frac{{\delta_{e} - \delta_{m} }}{2}} \right)} \right]^{2} \left( {\mu_{r}^{\prime } \cos \delta_{e} + \varepsilon_{r}^{\prime } \cos \delta_{m} } \right)^{2} }}$$

The small delta value and close to zero indicate good impedance matching. If |Δ| tends to far away from zero, it gives poor microwave absorption.

The MAMs can be roughly divided into three types according to their loss mechanisms including dielectric loss materials, magnetic loss materials and multiple loss materials, as shown in Table 1. Dielectric loss materials are represented by carbon materials [66, 67], non-magnetic metal powder [68, 69], polymers [70, 71], non-magnetic metal oxides [72, 73], non-oxygen ceramics [74, 75] and so on. They possess features such as high strength, resistance to high temperature, excellent electrical conductivity and low density, but effective absorption bandwidth (EAB) and the MA performance may be not sufficient [76, 77]. Magnetic loss materials are represented by magnetic metal powder and compounds [78, 79], ferrite [80–87], carbonyl iron [88–90] and so on. However, their high density and poor stability limit their practical application [91]. The dielectric and magnetic loss factors, defined as $\tan \delta_{E} = \varepsilon^{\prime \prime } /\varepsilon^{\prime }$ and $\tan \delta_{M} = \mu^{\prime \prime } /\mu^{\prime }$, are suggested to evaluate on dielectric and magnetic losses [92–94].

**Table 1:** Classification table of common MAMs

Types of MAMs	Typical materials	Loss mechanisms
Dielectric loss material	Carbon materials, non-magnetic metal powder, polymer, non-magnetic metal oxides, non-oxygen ceramics, etc.	Electrical conductivity loss, polarization relaxation loss (dipoles relaxation polarization and interfacial polarization)
Magnetic loss material	Magnetic metals and compounds, ferrite, carbonyl iron, etc.	Hysteresis loss, eddy current loss and residual loss
Multiple loss material	Combination of the above	Multiple loss

The dielectric loss ability mainly stems from electrical conductivity loss and polarization relaxation loss [95, 96]. The electrical conductivity loss is that when the EMWs enters into the MAMs, the charge carriers would form a current under the action of the electric field, and then, the electric energy converts to the thermal energy or other form of energy and dissipated out [97, 98], thus increasing EMW attenuation. However, if the conductivity is too high, the incident EMW will be reflected by a large amount, resulting in impedance mismatch and poor EMW attenuation. The polarization relaxation loss is split into ionic polarization, electronic polarization, dipoles relaxation polarization and interfacial polarization (spatial polarization) [55, 99]. Ion polarization is caused by the relative displacement of cations and anions. Electron polarization is caused by position change of the constituent atoms relative to the nucleus, and thus, the dipole moment is generated. Ion polarization and electron polarization usually occur in the frequency range of ultraviolet, visible and infrared light, which is much higher than the microwave frequency range (2-18 GHz), so they are excluded [100, 101]. Dipoles relaxation polarization refers to the polarization caused by the rotation of the dipole moment in the direction of the electric field, and it can greatly influence the dielectric loss [41, 102]. The relaxation loss can be analyzed by Debye equation [103–105]:

6 $$\varepsilon^{\prime } = \varepsilon_{\infty } + \left( {\varepsilon_{S} - \varepsilon_{\infty } } \right)\frac{1}{{1 + \omega^{2} \tau^{2} }}$$

7 $$\varepsilon^{\prime \prime } = \left( {\varepsilon_{S} - \varepsilon_{\infty } } \right)\frac{\omega \tau }{{1 + \omega^{2} \tau^{2} }}$$

We can deduce an equation from Eqs. (3) and (4) as follows:

8 $$\left( {\varepsilon^{\prime } - \frac{{\varepsilon_{S} + \varepsilon_{\infty } }}{2}} \right)^{2} + \left( {\varepsilon^{\prime \prime } } \right)^{2} = \left( {\frac{{\varepsilon_{S} - \varepsilon_{\infty } }}{2}} \right)^{2}$$

where $\varepsilon_{S}$ is the static dielectric constant, $\varepsilon_{\infty }$ is the dielectric constant of infinite frequency, and τ is the time of relaxation. The circle of this equation is called Cole–Cole semicircle [106, 107]. Each Cole–Cole semicircle represents a polarization relaxation process [108, 109]. The points on the semicircle correspond to the values of the real and imaginary parts of the dielectric constant at a certain frequency calculated by the Debye equation. Interfacial polarization usually appears at the interface of heterogeneous medium, which is caused by the accumulation of electrons or ions at the interface under the action of the external electric field [110]. Generally speaking, dielectric materials can be wideband absorption. However, the disadvantage is that the low-frequency absorption effect is poor and it is difficult to achieve the thin coating wideband absorption.

Magnetic loss refers to the phenomenon that the work is done by the outside world to a magnetic material and then the work is converted into heat during the process of magnetization or demagnetization [111]. It includes hysteresis loss, eddy current loss and residual loss [112, 113]. The hysteresis loss is due to the hysteresis loop relationship between the magnetic perceptual strength and the magnetic field strength. Normally, the hysteresis loss often occurred in the weak field can be excluded [114]. When a conductor moves in an inhomogeneous magnetic field or is in a time-varying magnetic field, the energy loss caused by the induced current in the conductor is called eddy current loss. It can be defined as [115–117]:

9 $$C_{0} = \frac{{\mu^{\prime \prime } }}{{\left( {\mu^{\prime } } \right)^{2} f}} = \frac{2}{3}\pi \mu^{0} \delta d^{2}$$

where δ is the electrical conductivity of material, and d is the thickness of the MAMs. From the equation, *C*_0_ is a constant at a certain thickness of the MAMs with the change of frequency. This is one of the ways to determine whether EMW loss only results from the eddy current loss [118]. The residual loss refers to other losses except hysteresis loss and eddy current loss [77, 101].

The multiple loss material is not just a single loss mechanism, and it combined the advantages of various losses.

We all know that the synergistic effects between the dielectric loss and magnetic loss contribute to the excellent EMW absorption ability, which result in the good impedance matching and strong EM wave attenuation of the MAMs. However, the conflict between the two sides is still exist. In order to get the EMW into the material as much as possible, it will inevitably reduce the attenuation ability of the MAMs to the EMW. Therefore, it is necessary to coordinate impedance matching and EMW attenuation in practical application. The attenuation constant α can be defined as [119, 120]:

10 $$\alpha = \frac{\sqrt 2 \pi f}{c}\sqrt {\left( {\mu^{\prime \prime } \varepsilon^{\prime \prime } - \mu^{\prime } \varepsilon^{\prime } } \right) + \sqrt {\left( {\mu^{\prime \prime } \varepsilon^{\prime \prime } - \mu^{\prime } \varepsilon^{\prime } } \right)^{2} + \left( {\mu^{\prime \prime } \varepsilon^{\prime \prime } + \mu^{\prime } \varepsilon^{\prime } } \right)^{2} } }$$

Dissipation of reflected EMW through the interference of MAMs is another key factor to attenuate the EMW, which can be described by the quarter-wavelength matching mechanism (*λ*/4) as follows [46, 109]:

11 $$d_{m} = \frac{n\lambda }{4} = \frac{nc}{{4f_{m} \sqrt {\left| {\mu_{r} } \right|\left| {\varepsilon_{r} } \right|} }}\left( {n = 1, \, 3, \, 5} \right)$$

In the formula, *λ* is the wavelength of EMW, *d*_*m*_ and *f*_*m*_ are the thickness and corresponding frequency of maximum RL values, and *|μ*_*r*_| and *|ε*_*r*_| are the modulus of complex permeability and permittivity at *f*_m_, respectively.

## MOF-Derived PC-Based Nanocomposites as MAMs

As we know, MOFs are composed of inorganic metal center (metal ion or metal cluster) and organic ligand [121, 122]. Through direct pyrolysis of MOFs, they can be converted into metal-doped carbon, and the structure does not change significantly. We can simple and fast synthesis of MAMs by pyrolysis. However, the pure MOF-derived PC-based nanocomposites have not been functionalized, the absorption loss mechanism is simple and MA performance may be not exciting. By incorporated with materials with different absorbing loss mechanisms, impedance matching can be improved and the MA performance can be enhanced.

### Magnetic Single-Metal MOF-Derived PC-Based Nanocomposites as MAMs

Common magnetic metals are Fe, Co and Ni. They often act as inorganic metal centers to synthesize MOF. When they are directly pyrolyzed, the MA performance may not be very good because the simple loss mechanism may lead to impedance mismatch. They often coupled with dielectric loss material to improve impedance matching and rational design on the microstructure to introduce multiple loss mechanisms.

#### Co-MOF-Derived PC-Based Nanocomposites as MAMs

**Fig. 2:**
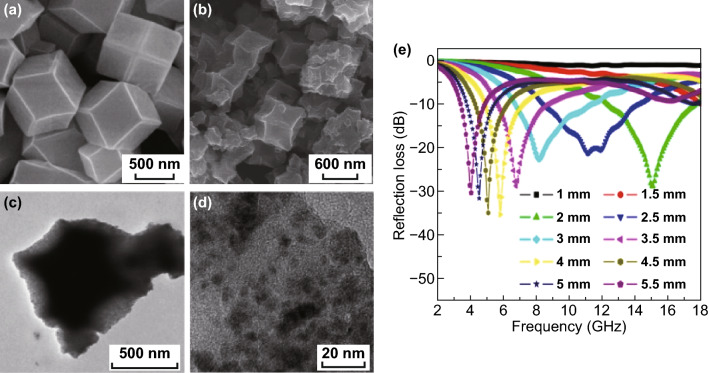
**a**, **b** SEM images of Co/C before and after calcination at 500 °C under Ar for 5 h. **c**, **d** Low and high magnification TEM images of Co/C-500. **e** Calculated results of the reflection loss vs frequency for Co/C-500 with different thicknesses. Reprinted with permission from Ref. [123]

The most widely studied MOF-derived PC-based nanocomposites as MAMs are the Co-MOF. The typical Co-MOF is ZIF (zeolitic imidazolate framework)-67, which is prepared through the self-assembly of Co^2+^ and 2-methylimidazole. In Kuang’s work, they pyrolyze Co-based MOFs (Co-MOF, ZIF-67) to synthesize porous Co/C composite under inert atmosphere with different pyrolysis temperature [123]. The morphology before and after sintering has not changed much, just the surface was wrinkled, as shown in Fig. 2. The sample pyrolysis at 500 °C shows better MA performance. The maximum *RL* of Co/C-500 reached − 35.3 dB at 5.8 GHz with a thickness of 4 mm, and the *EAB* was 5.80 GHz (8.40–14.20 GHz) corresponding to a thickness of 2.5 mm. The magnetic loss of Co, the large dielectric loss value of carbon and the porous structure result in the MA performance, but the *RL* and *EAB* are not very satisfactory.

Since the tunable of inorganic metal center and organic ligand, MOFs have various of compositions and structures. Co-MOF-derived PC-based nanocomposites as MAMs have been reported by many groups. Kong synthesized Co/C pyrolysis from the cubic [Co(INA)_2_]MOF by isonicotinic acid as organic ligand [124]. Wang’s group constructed Co/C composites via pyrolysis a new Co-based MOF named [Co_2_O(cptpy)_2_(DMF)] (CPT-1-Co, Hcptpy = 4′-(4-carboxyphenyl)-4,2′:6′,4′′-terpyridine, DMF = N,N-dimethylformamide), by reacting a multi-dentate ligand, 4′-(4-carboxyphenyl)-4,2′:6′,4′′-terpyridine (Hcptpy), with Co(OAc)_2_ salt [125]. More works of Co/C synthesized from pure Co-MOF have also been reported [126, 127]. However, the MA performance of pure Co-MOF-derived PC-based nanocomposites is not quite satisfying. The reason may be the low relatively complex permittivity, and high relatively complex permeability leads to poor impedance matching performance, thus limiting their application in MAMs. So, Co-MOF coupled with other loss material, especially dielectric loss material, is a better solution to improve the MA performance.

When they coupled with MOF-derived PC, they would show unexpected performance. The MOF-derived PC/dielectric loss material nanocomposites have many advantages, such as low cost, easy preparation and low density. Moreover, the additional loss mechanisms are created, and electrical loss, polarization loss, the interfacial and multiple scattering may lead to the good MA performance.

**Fig. 3:**
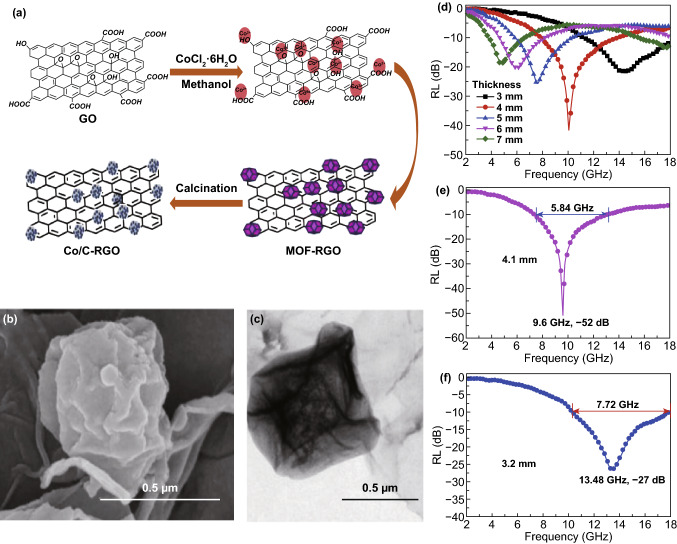
**a** Process for the preparation of MOF/RGO. **b**, **c** SEM and TEM images of heat-treated MOF/GO hybrid at 500 °C for 6 h. **d–f** RL curves of MOF/GO-500 and under 3.2 and 4.1 mm with a filler loading ratio of 6 wt% in wax. Reproduced with permission from Ref. [128]

Carbon materials such as graphene, carbon nanotube (CNT) and carbon nanofiber (CNF) have aroused wide attention as MAMs due to their excellent physical and chemical properties, including their lightweight, high specific surface area, mechanical strength, thermal stability, corrosion resistance, electric conductivity and dielectric properties. Graphene, composed of *sp*^2^-bonded carbon atoms, has a lot of advantages such as electrical, thermal and mechanical properties. Graphene has functional groups and some defects on its surface. The impedance matching of graphene can be improved, and the dipole polarization relaxation can also be generated to improve the MA performance. Graphene can also form a multilayer structure, which can increase the number of reflections and propagation distance of EMW. Dong had synthesized MOF/RGO hybrids by two steps including in situ growth of Co-based MOF on GO nanosheets and a controlled calcination process [128], which is shown in Fig. 3. The maximum *RL* of the sample reached − 52 dB at 9.6 GHz with a thickness of 4.1 mm. The *EAB* of this MOF/RGO hybrid can reach 7.72 GHz only under a thickness of 3.2 mm, which surpasses most reported MOF and RGO-based MAMs. It is worthy to point out that the enhanced effect of MOF/RGO interface, improved match between dielectric loss and magnetic loss should be considered as the factor of the high MA performance.

Chen’s group synthesized CoC–rGO obtained by calcination of ZIF-67–GO hybrids [129]. The introduction of high conductivity of rGO may lead to strong eddy current loss and reduce the permeability. The impedance matching is determined by the additive amount of rGO. The *RL*_max_ value reached up to -44.77 dB at thickness of 2.1 mm, and the *EAB* reached 5.2 GHz at thickness of 1.8 mm, which showed evident advantages compared to CoC or rGO alone with single loss mechanism. Zhang’s group synthesized MOF-derived carbonaceous Co_3_O_4_/Co/RGO composite at 600 °C in Ar [130]. The sample displays *RL*_max_ − 52.8 dB at 13.12 GHz with thickness which is only 2.0 mm. The *EAB* is up to 10.72 GHz in the thicknesses range of 2.0–4.0 mm. Since the unique porous structure, the dielectric and magnetic tangent losses of the sample are in the middle levels, it is beneficial for the impedance match and thus results in the higher MA performances. CNTs can be divided into single-walled carbon nanotubes (SCNTs) and multi-walled carbon nanotubes (MCNTs). They have a very large aspect ratio, so a conductive network can be formed. The dielectric constant is large, and its permeability is small, so its impedance matching is poor. Therefore, it is often combined with other magnetic loss materials to improve the MA performance. Dong et al. had prepared a 3D Co/C-MCNTs hybrid network using MCNTs as wires and Co-based MOFs as junctions [131]. The multiple components synergistic effect leads to the good MA performance. The purpose of introducing MCNTs may promote the formation of a conductive network, increasing interfacial polarization. The *RL*_max_ of the sample Co/C-MCNTs is -33.4 dB at the frequency of 3.6 GHz with the thickness of 6 mm. And the EAB is 4.08 GHz at 1.8 mm. Yu had also fabricated Co–C/MWCNTs composites, and the *RL*_max_ is − 48.9 dB at 2.99 mm [132]. Tan synthesized ultra-small Co/CNTs nanohybrid via the pyrolysis of ZIF-67 and (catalytic chemical vapor deposition) CCVD method. It achieves the *RL*_max_ of − 49.16 dB and the in EAB of 4.2 GHz (12.4–16.6 GHz) [133]. Chen synthesized MWCNTs@carbonaceous CoO composites with good MA properties [134]. When the annealing temperature is 500 and 600 °C, the carbonaceous Co_3_O_4_ can be obtained. When the annealing temperature is 700 °C, Co_3_O_4_ was all reduced to CoO. The value of *RL*_max_ is up to − 50.2 dB with 1.84 mm thickness. CNF has high dielectric constant, so the impedance match may be not very good, and we usually improve the MA performance by combining with magnetic loss materials. Zhang et al. reported necklace-like CNFs@MOF-based carbonaceous Co/CoO composite, which was synthesized by wet and pyrolysis method [135]. The optimum *RL* value is − 53.1 dB at 6.56 GHz with the thickness 3.54 mm, and *EAB* is up to 13.52 GHz with the thickness range of 2.0–5.0 mm. The unique structure will form many defects, which can generate much interfacial polarization, which lead to more dielectric loss. The small-sized nanoparticle can improve dipole polarization. And impedance matching is also optimized to improve the MA performance.

Polymer has the advantages of low density, anti-corrosion and adjustable conductivity, and the electric conductivity and dielectric constant are high. So, it has shown promising prospect in the field of MAMs. In Wang’s report, a chain-like PPy (Polypyrrole) aerogel decorated with MOF-based nanoporous Co/C (Co/C@PPy) has been successfully prepared by a self-assembled polymerization method [136]. The composite Co/C@PPy can reach the optimal *RL* value of − 44.76 dB at 17.32 GHz with the thickness of 2.0 mm. And the *EAB* of 6.56 GHz (11.04–17.60 GHz) is achieved with the thickness of 2.5 mm. The performance is attributed to a proper impedance matching and a high dielectric loss highly enhanced by the PPy aerogel. Besides, the unique chain-like PPy aerogel and the porous feature of Co/C itself can induce more multiple reflection and scattering of EMW.

Non-magnetic metal oxide is a common dielectric loss material and coupled with magnetic loss material to regulate impedance matching which is an effective strategy to solve the absorption problems. Zinc oxide (ZnO), as an important semiconductor with a wide band gap, has been extensively investigated as MAMs, due to its excellent dielectric properties and lightweight [137]. Hu’s group constructed a novel 3D hetero-structured Co/NPC@ZnO/rGO by the direct pyrolysis of ZIF-67@ZnO NPs wrapped on rGO nanosheets [138]. ZnO can be utilized to regulate the complex permittivity over the measured frequency range and upgrade the impedance matching property of the sample. The *RL*_max_ can reach up to − 45.4 dB at only 2 mm, and the *EAB* achieved 5.4 GHz (from 11.9 to17.3 GHz). Vanadium sesquioxide (V_2_O_3_), with relatively high electrical conductivity at room temperature and superior dielectric loss, is usually used in the field of MAMs. Yan’s group designed and synthesized Co/C@V_2_O_3_ hollow spheres with an *RL* of − 40.1 dB and the *EAB* of 4.64 GHz at a small thickness of only 1.5 mm [139]. The sample exhibits both excellent impedance matching and light weight due to the rational combination of hollow V_2_O_3_ spheres and porous Co/C. One-dimensional chain-like MnO@Co/C composite derived from MnO_x_@ZIF-67 has also been reported [140]. Similar work was also reported by Co/N/C@MnO_2_ sample [141]. Hierarchical MnO_2_ sheets are used to decrease the excessive complex permittivity of Co/N/C and improving impedance match, and polydopamine (PDA) is carbon source. The sample with a filler loading of 15 wt% shows the *RL*_max_ of − 58.9 dB and *EAB* of 5.5 GHz. The excellent MA performance of Co/N/C@MnO_2_ composites is attributed to synergetic effects of excellent impedance match, and dramatical EM attenuation ability arises from multiple helpful constituents, abundant interfaces and extraordinary hollow structure.

Non-oxygen ceramics presents high strength, good thermal stability and chemical resistivity. But the MA performance is not very good. In order to improve the MA performance, we usually combine non-oxygen ceramics with magnetic loss materials. Dong constructed kebab-like nanocomposites composed of SiC stringing polyhedral Co-MOF [142]. The excellent MA performance attributed to the reduced dielectric constant, enlarged aspect ratio and enhanced interface polarization. Under the thickness of 3 mm, the *RL*_max_ attained − 47 dB at the frequency of 9.32 GHz, and the *EAB* achieved 5.92 GHz in a frequency range 12.08–18 GHz with the sample thickness of 2.0 mm.

**Fig. 4:**
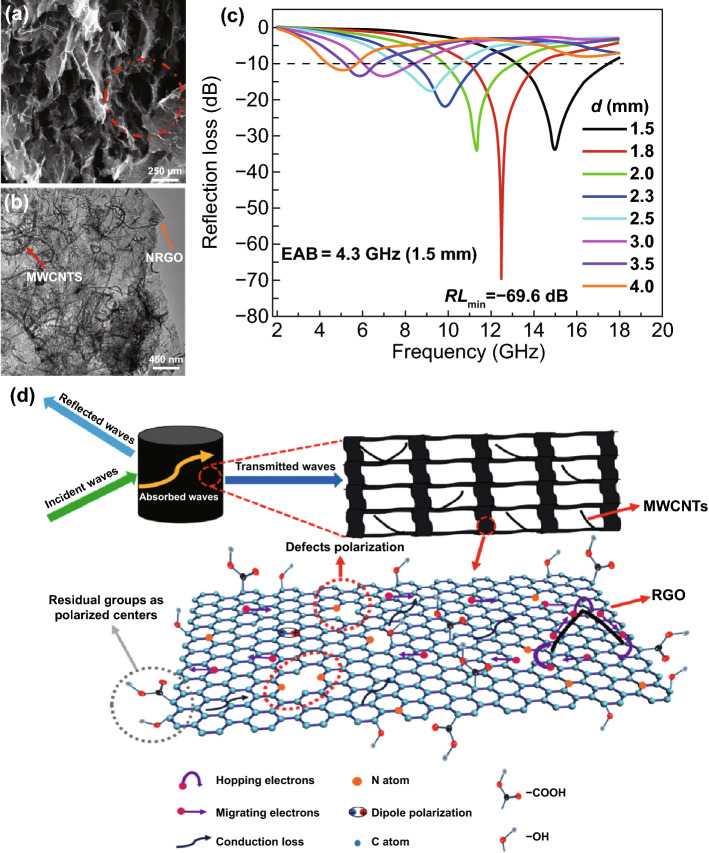
**a**, **b** SEM and TEM images of the samples. **c** RL-f curves of the sample. **d** Schematic diagram of the EM absorbing mechanisms of NRGO/MWCNT composite foams. Reprinted with permission from Ref. [143]

Rational design on the microstructure of MOFs-derived PC-based nanocomposites is an effective strategy to prepare high-performance MAMs, through designing some special structures such as foam structure, core–shell structure and hollow structure, which improve the multiple reflections and interfacial polarization so as to enhance MA performance. Zheng’s group successfully synthetized NRGO (nitrogen-doped)/MWCNT composite foams by hydrothermal and high-temperature calcination strategy [143]. The 3D networks were well constructed by overlapped flaky RGO in the composite foams, and the calcination temperature showed notable effects on the micromorphology. The *RL*_max_ is − 69.6 dB at 12.5 GHz, and EAB achieved 4.3 GHz (13.2–17.5 GHz) at a low thickness of 1.5 mm, as shown in Fig. 4. The excellent MA performance of the foams was derived from a well-constructed 3D network structure, nitrogen doping, polarization relaxation and conduction loss. Wu and his workmates designed a 3D hybrid carbon sponge composite with a hierarchical micro/nanostructure and hollow skeleton [144]. The conductive network, diverse interface, porous and tubular structures, as well as the synergistic effect between metallic Co nanocrystals and carbon species, resulting in the *RL*_max_ is − 51.2 dB with a ultrathin thickness of 1.6 mm and *EAB* is 5.4 GHz. MOFs-derived hollow Co/C microspheres were produced by Li’s group [145]. They use cetyltrimethylammonium bromide (CTAB) as self-sacrificing template to make hollow microstructures well preserved in the resultant carbon matrix. The hollow microstructures improve the dielectric loss, magnetic loss and enhancing attenuation ability. So rational design on the microstructure of MOFs-derived PC nanocomposites is an effective strategy to develop high-performance MAMs.

#### Ni-MOF-Derived PC-Based Nanocomposites as MAMs

**Fig. 5:**
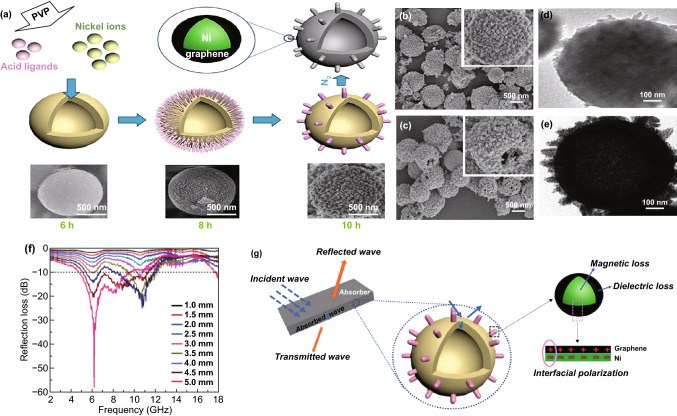
**a** Illustration for the formation of Ni-MOF hollow spheres with controllable surface architecture. **b**, **c** SEM images and **d**, **e** TEM images of Ni-MOFs samples with 10 h before and after annealing at 600 °C. **f** Electromagnetic wave reflection losses of the Ni-MOF sample with different reaction times. **g** Absorbing mechanism of as-prepared samples. Reprinted with permission from Ref. [148]

Magnetic metal Ni-based MOF is also often used in MAMs. Zou and his workmates synthesized Ni@C composites by thermal decomposition of pure Ni-MOF [146]. The sample which calcinated at temperature 800 °C showed a *RL*_max_ of − 55.7 dB, and the *EAB* is 6.0 GHz at a thickness of 1.85 mm. The excellent MA performance is related to the hollow structure and the synergistic effect between carbon and nickel nanoparticles. Ji had fabricated Ni nanoparticles-embedded nanoporous carbon (NPC/Ni), and the sample prepared at 700 °C exhibits nice MA performance with the *RL*_max_ value of -39.4 dB and *EAB* of 4.2 GHz [147]. In Yang’s report, they synthesized Ni-based MOF hollow spheres with various surface morphologies via a simple hydrothermal method [148]. The surface morphologies are controlled by the hydrothermal reaction time. The surface morphologies are smooth, hair-like and rod-like corresponding to the reaction time which is 6, 8, and 10 h, respectively. The *RL*_max_ of the 10 h sample reached − 58 dB at 6 GHz with a thickness of 1.5 mm, and the *EAB* was 6.2 GHz (5–11.2 GHz) with a thickness of 4.6 mm, as shown in Fig. 5. The difference in the surface morphologies results in variation of the magnetic anisotropy, which leads to multi-resonance behavior of the permeability. Liu’s group synthesized two kinds of Ni@C derived from the Ni-based MOFs with two kinds of organic ligands (dimethylimidazole as a ligand named as Ni-ZIF and trimesic acid as a ligand named as Ni-BTC) [149]. The *RL*_max_ of the Ni@C-ZIF microspheres is − 86.8 dB with the thickness of 2.7 mm, and the *EAB* was 7.4 GHz (4–11.4 GHz) with the thickness ranging from 1.5 to 4.0 mm. The impedance matching, multiple reflection, interfacial polarization among Ni and C and the N-doping were beneficial to the excellent MA performance.

**Fig. 6:**
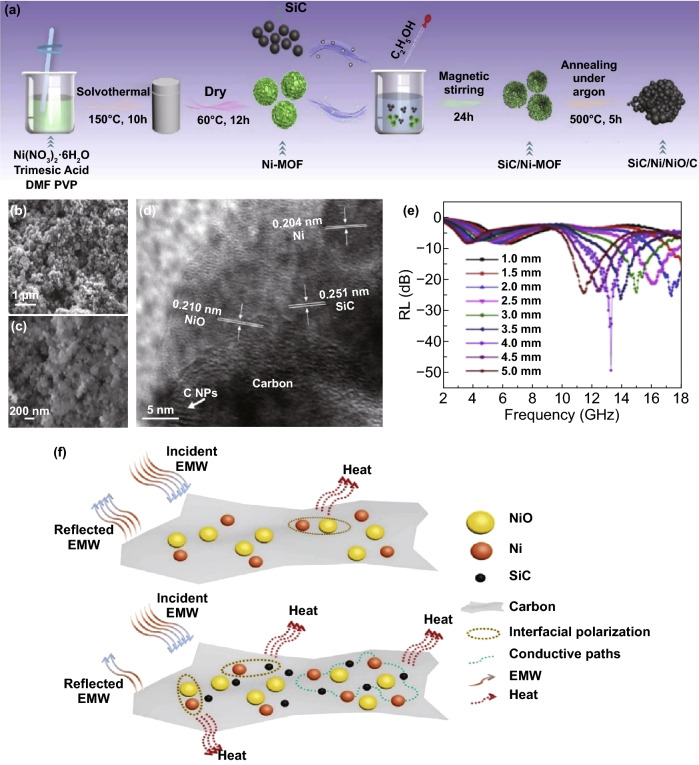
**a** Preparation of the SiC/Ni/NiO/C nanocomposites. **b**, **c** SEM images and **d** HRTEM image of the SiC/Ni/NiO/C nanocomposites. **e** RL curves of SiC/Ni/NiO/C with a filler loading of 20 wt% in paraffin. **f** Schematic of the electromagnetic wave (EMW) attenuation mechanisms of Ni/NiO/C and SiC/Ni/NiO/C nanocomposites. Reprinted with permission from Ref. [150]

The dielectric loss material is also usually introduced to Ni-MOF to improve impedance matching. Chen’s group fabricated multi-component composite SiC/Ni/NiO/C by annealing SiC NPs and the Ni-MOF in argon [150]. The maximum *RL* is − 50.52 dB at 13 GHz for a film thickness of 4.0 mm, and the *EAB* is 2.96 GHz (14.76–17.72 GHz) with thickness of 2.5 mm. The high permittivity of the SiC/Ni/NiO/C nanocomposites is expected to enhance absorption of EMW, as shown in Fig. 6. The excellent MA performance also stems from the multi-interface structure which provides interfacial polarization and plasmon resonance.

Structure design is also very important to improve the MA performance of Ni-MOF-derived PC. Du synthesized hierarchical yolk–shell nanostructure (NiO/Ni/GN@Air@NiO/Ni/GN) derived from Ni-MOF by solvothermal reactions [151]. This special structure can effectively enhance the MA performance. And it can also tune the dielectric properties of the NiO/Ni/GN@Air@NiO/Ni/GN composites to achieve good impedance matching. The *RL*_max_ of − 34.5 dB is obtained at 17.2 GHz with the thin thickness of 1.7 mm. And the *EAB* can be obtained in the frequency range 7.8–18 GHz with absorber thicknesses of 1.7–5.0 mm. The 3D porous flower-like Ni/C composites were prepared by Zou’s group through the pyrolysis of Zn-doped Ni-MOF under N_2_ atmosphere [152]. These 3D flower-like structures have massive porous and large spacing flakes, which increases the EMW scatter. The *RL*_max_ is − 52.4 dB with a thickness of 1.6 mm, and *EAB* is 5 GHz.

#### Fe-MOF-Derived PC-Based Nanocomposites as MAMs

**Fig. 7:**
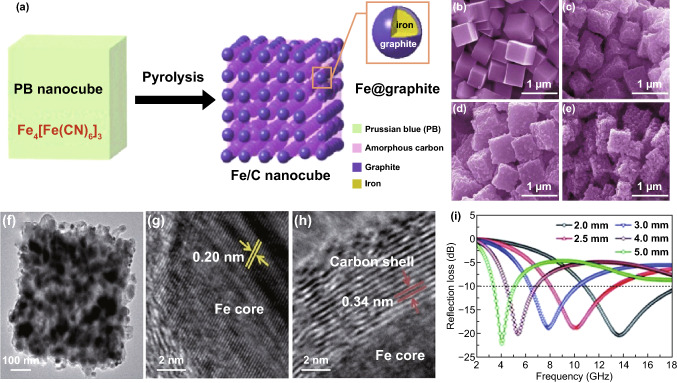
**a** Schematic illustration of converting PB nanocubes into Fe/C nanocubes by a pyrolysis technique. **b–e** SEM images of the as-prepared PB nanocubes and Fe/C nanocubes obtained at different pyrolysis temperatures: 600, 650 and 700 °C. **f** TEM image of the 650 °C sample. **g** HRTEM images of the Fe core and **h** the graphitic carbon shell. **i** Reflection losses of the 650 °C sample with variable absorber thicknesses. Reprinted with permission from Ref. [153]

Because of the good chemical stability, high saturation magnetization and simple preparation, metal iron and ferrite are often used to enhance magnetic loss in MA. So dielectric loss material is also introduced to improve impedance matching. Xu et al. reported Fe/C nanocubes, which are prepared through an in situ derivation from Prussian blue MOF by controlled high-temperature pyrolysis [153]. The maximum *RL* of the sample obtained at 650 °C reached − 22.6 dB at 4 GHz with a thickness of 5 mm, and the EAB was 7.2 GHz (10.8–18.0 GHz) corresponding to a thickness of 2 mm, as shown in Fig. 7. The good MA performance of the Fe/C nanocubes results in the synergetic effect of dielectric loss and magnetic loss.

Hu’s group successfully fabricated magnetic Fe_3_C/C (denoted as FC-650) and Fe_3_C/Fe/C (denoted as FC-700) carbon-matrix composites via carbonization of Material Institute Lavoisier (MIL)-101(Fe) [154]. Both Fe_3_C/C and Fe_3_C/Fe/C owned flower-like structures formed by 2D flakes. Fe_3_C/C possessed *RL*_max_ of − 39.43 dB at 14.00 GHz, at the thickness of 2.00 mm. And the *EAB* is 14.32 GHz (from 3.68 to 18.00 GHz). The impedance matching and the well-designed structures lead to the excellent MA performance.

The morphology usually had significant effect on the MA performance. Kong pyrolyzed two MOFs with different topologies (MOFs: MIL-101-Fe and MIL-88B-Fe) under same pyrolysis condition, identical chemical composition and microstructure [155]. The *RL*_max_ is − 59.2 dB with a thickness of 4.32 mm, and the *EAB* is 6.5 GHz with a thickness of 2 mm which are achieved by Fe/C-700@101 (700: pyrolysis temperature; 101: MIL-101 precursor) and Fe/Fe_3_C/C-800@101, respectively. This article reveals the significant impact of morphology on MA performance.

**Fig. 8:**
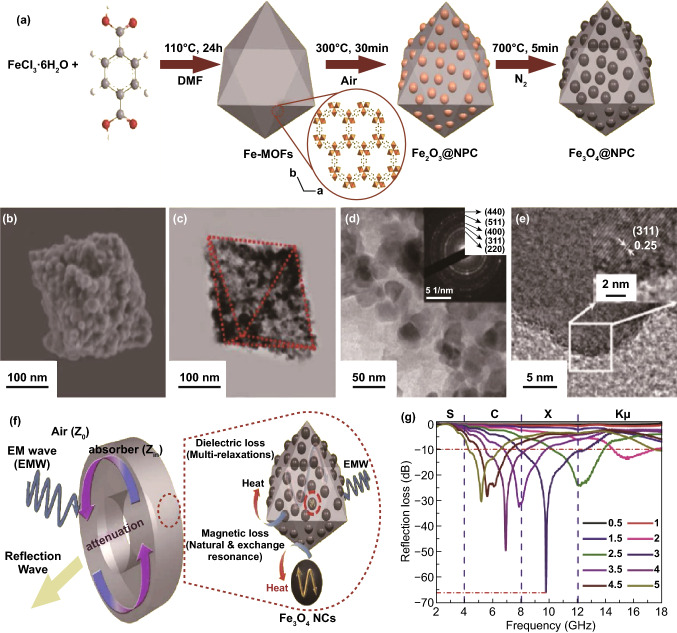
**a** Schematic illustration of the Fe_3_O_4_@NPC composites formation process. **b–e** SEM, low magnification TEM images, high magnification TEM images (inset: SAED patterns) and HRTEM images of Fe_3_O_4_@NPC composites. **f** Schematic illustration of the electromagnetic wave absorption mechanism. **g** Electromagnetic wave reflection loss with various thicknesses for Fe_3_O_4_@NPC composites. Reprinted with permission from Ref. [157]

In order to improve the impedance matching, dielectric loss material is usually introduced to the Fe-MOF. Hu’s group reported the synthesis of novel MOF (Fe)/PANI (polyaniline) core–shell composite via hydrothermal and in situ chemical polymerization methods [156]. The *RL*_max_ of the composite can reach − 41.4 dB at 11.6 GHz, and the *EAB* is up to 5.5 GHz with only 2 mm. The loss mechanism is due to the enhanced interfacial polarization, dipole polarization and charge transfer and attenuation constant. The introduction of PANI enlarges the dielectric constant. Meanwhile, the higher dielectric constant of MOF (Fe)/PANI may be attributed to the improved interfacial polarization and appearance of localized defects as bipolaron/polaron, and the multiple reflection and scattering in the pores of the samples, dielectric loss, magnetic loss, good impedance matching also beneficial for EMW absorption. Lu’s group reported Fe_3_O_4_ @ carbon (Fe_3_O_4_@NPC) composites by a simple one-pot synthesis method and subsequent in situ formation under thermal decomposition conditions [157]. The Fe_3_O_4_@NPC composites exhibited MA performance with a maximum *RL* of -65.5 dB at 9.8 GHz with a thickness of 3 mm and the *EAB* of 4.5 GHz, as shown in Fig. 8. The tan δ_m_ value was higher than tan δ_ε_ value, which indicated that magnetic loss contributed more than dielectric loss to the EMW attenuation. Thus, the improvement of the absorption performance was mainly originated from the magnetic loss. The synergistic effects of the dielectric loss and the magnetic loss are effective in enhancing the MA performance.

### Non-magnetic Single-Metal MOF-Derived PC-Based Nanocomposites as MAMs

Non-magnetic single-metal MOF-derived PC-based nanocomposites usually act as dielectric performance but have negative characteristics in attenuation and impedance matching. Therefore, selecting a high dielectric candidate to combine with non-magnetic single-metal MOF or magnetic loss material is critical.

#### Zn-MOF-Derived PC-Based Nanocomposites as MAMs

**Fig. 9:**
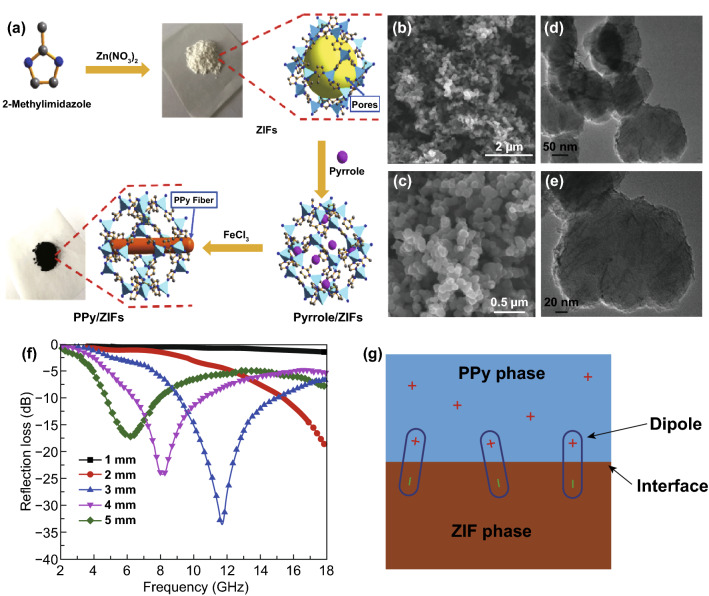
**a** Preparation route of PPy/ZIFs nanocomposites. **b**, **c** SEM images, **d**, **e** TEM images and **f** MA performance of PPy/ZIFs. **g** Interfacial polarization of interfacial polarization. Reprinted with permission from Ref. [160]

Ji’s group fabricated ZnO/nanoporous carbon (NPC)/reduced graphene oxide (RGO) materials through a simple and valid hydrothermal method derived from Zn-MOF [158]. The *RL*_max_ is − 50.5 dB with a thickness of 2.4 mm, and the *EAB* is 7.4 GHz with a thickness of 2.6 mm, which is shown in Fig. 9. The dielectric constant of ZnO/NPC/RGO samples could be modulated by regulating the combination ratio. Too high or too low permittivity can hardly satisfy an ideal absorber. They had also prepared novel ZnO/carbon porous nanofibers derived from Zn-MOF and polyacrylonitrile (PAN) nanofibers [159]. Xie reported polypyrrole (PPy)/Zn-MOF nanocomposites show tunable electrical conductivity as well as a tunable MA performance [160]. The EAB reaches 7.24 GHz with the thickness of 2.6 mm, and the *RL*_max_ is -49 dB with the thickness of 2.9 mm. The MA performance is attributed to the electrical conduction loss and interfacial polarization relaxation.

#### Ti-MOF-Derived PC-Based Nanocomposites as MAMs

Ji’s group had also synthesized a novel nanoporous carbon material (TiO_2_/C) by annealing titanium-based MOFs (MIL-125 (Ti); MIL stands for Material from Institute Lavoisier) [161]. The *RL*_max_ is -49.6 dB, and the EAB is 4.6 GHz (13.4-18 GHz) with the thickness of 1.6 mm. The outstanding MA performance may be due to the high tan δ_ε_, α and polarization loss.

#### Cu-MOF-Derived PC-Based Nanocomposites as MAMs

Zeng’s group synthesized Ni/NiO/Cu@C composites by using Cu MOFs as the precursor [162]. The *RL*_max_ value is − 38.1 dB at a layer thickness of 3.2 mm. The introduction of Ni offers magnetic loss, and interfacial polarization is changed by increasing the interface area and electrical conductivity.

#### Zr-MOF-Derived PC-Based Nanocomposites as MAMs

Liu’s group developed cobalt-decorated porous ZrO_2_/C hybrid octahedrons by pyrolysis of Co(NO_3_)_2_ impregnated NH_2_-UIO-66(Zr-MOF) [44]. The sample results in *RL*_max_ of − 57.2 dB at 15.8 GHz, corresponding to a matching thickness of 3.3 mm. The *EAB* reaches 11.9 GHz (6.1–18 GHz). The excellent MA performance of Co/ZrO_2_/C can be ascribed to the strong interface polarization and the suitable impedance matching, and the synergistic effect among the components. Wang had also synthesized ZrO_2_/C octahedra from UIO-66 [163]. The *RL*_max_ value of − 58.7 dB (16.8 GHz, 1.5 mm) has been achieved. And the EAB could cover 91.3% (3.4–18.0 GHz) of the measured frequency within the thickness range of 1.0–5.0 mm.

#### Rare-Earth MOF-Derived PC-Based Nanocomposites as MAMs

Li’s group had reported the synthesis of a series of rare-earth MOFs based on MH (maleic hydrazide) ligands [13]. RE-MOFs have many advantages such as hierarchical porous structures, low density and large pore volume. These properties will meet the requirements of MAMs. They successfully synthesized four novel RE-MOFs [Y_2_(MH)_6_]_n_·DMF (1), [Er_2_(MH)_6_]_n_ (2), [Yb_2_(MH)_6_]_n_ (3) and [La(MH)_3_]_n_ (4) by the traditional hydrothermal method. Different MA performances can be attributed to different structures and different central ions. The maximum *RL* values of MOF 1, MOF 2, MOF 3 and MOF 4 are − 22.78 dB at 5 mm, − 19.99 dB at 4.5 mm, − 28.14 dB at 2 mm and − 13.07 GHz at 2 mm, respectively. And the effective absorption bandwidth is 2.24 GHz (6.8–9.04 GHz), 2.12 GHz (from 6.8 to 8.72 GHz), 0.96 GHz (15.76–16.72 GHz) and 0.32 GHz (16.72–17.04 GHz) for MOF 1, MOF 2, MOF 3 and MOF 4. The property may be resulted in the synergetic effects of permittivity and permeability.

### Multi-metal MOF-Derived PC-Based Nanocomposites as MAMs

The MA performance of multi-metal MOF-derived PC-based nanocomposite is often better than single-metal MOF because the multi-metal MOF combines the advantages of two or more materials, endows the mixture with new chemical and physical properties and effectively regulates the electromagnetic parameters of the MAMs.

#### Multi-magnetic Metal MOF-Derived PC-Based Nanocomposites as MAMs

**Fig. 10:**
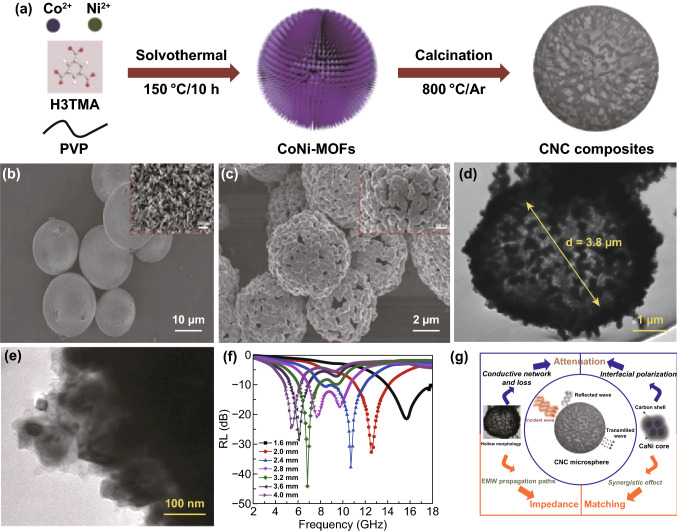
**a** Illustration for the synthetic process of hollow CNC microspheres. **b**, **c** SEM images, **d**, **e** TEM images and **f** RL curves of CoNi@C samples. **g** Schematic illustration of microwave absorption mechanisms for CNC microspheres. Reprinted with permission from Ref. [165]

NiCo nanoparticles/nanoporous carbon (NiCo/NPC) composites with multilayered structure were synthesized through in situ pyrolysis of the bimetallic NiCo-MOF by Lu’s group [164]. The synergistic interactions of magnetic loss and dielectric loss among NiCo NPs, graphitized carbon layer and NPC were beneficial to the optimization of the impedance matching and the enhancement of EMW attenuation. The multilayered nanoporous carbon matrix leads to the multiple reflection and scatterings, interface and dipole polarization as well as the natural resonance and exchange resonance. The *RL*_max_ value is − 51 dB at 17.9 GHz with *EAB* of 4.5 GHz (13.5–18 GHz) and a thickness of 1.5 mm at 600 °C. Dong had also fabricated porous and hollow CoNi@C microspheres derived from CoNi-MOFs [165]. The *RL*_max_ can reach − 44.8 dB at 10.7 GHz, and the *EAB* can reach up to 13.3 GHz (4.7–18.0 GHz) with the thickness of 1.6–4.0 mm, as shown in Fig. 10. The simultaneous enhancement of attenuation ability and impedance matching together contribute to the improved MA performance. The attenuation ability comes from interfacial polarization, eddy current loss, multiple reflection and scattering. The impedance matching stems from magnetic CoNi alloy and dielectric graphitized carbon. Liu had also reported CoNi/C nanocomposites derived from bimetallic CoNi-MOF [166]. The *RL*_max_ of − 74.7 dB could be achieved with a thickness of 1.8 mm at 15.6 GHz. The *EAB* ranged from 2.9 GHz to 18 GHz. The porous Co–Ni/C nanocomposites combined advantages of excellent impedance matching and strong interfacial loss between metallic NPs and porous carbon composites. Similarly, FeCo alloy/carbon composites [167] and Fe_3_Ni/C composites [168] had also shown the excellent MA performance. Hollow sphere trimetallic FeCoNi@C MAMs via high-temperature carbonization were obtained using FeCoNi-based MOF-74 (FeCoNiMOF) as the precursor [169].

Other multi-magnetic metal MOF-derived PC-based nanocomposites as MAMs had also been reported. In order to further improve impedance matching, the dielectric loss is often introduced to the multi-magnetic metal MOF such as FeCo@C@CNGs(carbon nanocages) [170], NiCo alloy/carbon nanorod@CNT [171], Fe–Co/NC/rGO [172], FeNi@CNT/CNRs(carbon nanorods) [173] and CoFe@C@MnO_2_ [174]. All of these samples show good impedance matching and outstanding EMW attenuation capability.

#### Magnetic and Non-magnetic Metal MOF-Derived PC-Based Nanocomposites as MAMs

**Fig. 11:**
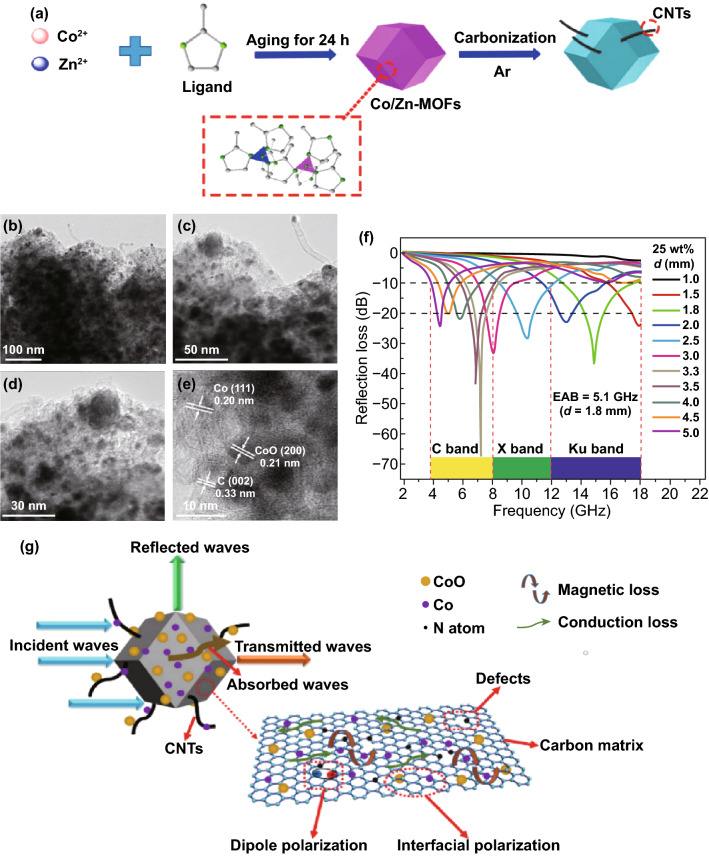
**a** Schematic illustration of the preparation procedures of CoO/Co/C nanocomposites. **b–e** TEM images with different magnifications of CoO/Co/C nanocomposites. **f** RL curves of CoO/Co/C nanocomposites. **g** Schematic illustration of the possible microwave absorption mechanisms of nitrogen-doped CoO/Co/C nanocomposites. Reprinted with permission from Ref. [175]

Non-magnetic metal MOFs play the role of dielectric loss. Zn is most widely used in this occasion. Since the unique evaporation character of Zn metal under high pyrolysis temperature, the porous low-dielectric amorphous carbon/Zn shell derived from Zn-MOF was formed to decrease the permittivity for a better impedance match. Zheng’s group fabricated nitrogen-doped CoO/Co/C nanocomposites by high-temperature pyrolysis of Co/Zn-ZIFs [175]. Zn was evaporated during the high-temperature pyrolysis process at 700 °C. The *RL*_max_ reached − 66.7 dB at 7.2 GHz with a thickness of 3.3 mm, and the *EAB* is 5.1 GHz (12.6–17.7 GHz) with thickness of 1.8 mm, as shown in Fig. 11. The excellent MA performance ascribed to the enhanced polarization relaxation, and synergistic effects of dielectric loss, conduction loss and magnetic loss.

Jiang had fabricated CoZn-MOF and then calcined it at different high temperatures to gain the metal Co embedded in porous and N-doped graphitized carbon matrix (Co@pNGC) [176]. Zn species was also evaporated at high temperature. The *RL*_max_ is − 50.7 dB at 11.3 GHz, and the *EAB* reaches 5.5 GHz (12.3–17.8 GHz), corresponding to a thickness of 2.0 mm. The strong dielectric loss is derived from interfacial polarization, migration, hopping of electrons and the magnetic loss from the Co nanoparticles.

## Comparison of MA Performance of Different MOF-Derived PC-Based Nanocomposites

As is mentioned above, many MOF-derived PC-based nanocomposites exhibited the appreciable MA performance. In Table 2, we sum up the performance of the MAMs mentioned above. As is described, there are many kinds of MOF-derived PC-based materials used in the MAMs. Most of the MOF-derived PC-based MAMs have better MA performance than the comparison MAMs. The MA performance of pure Ni, Co, Fe-MOF-derived PC-based is not satisfactory. When they coupled with dielectric loss material, the MA performance will be significantly improved. Non-magnetic metal MOF-derived PC-based MAMs such as Zn, Ti, Cu, Zr and RE are usually coupled with magnetic loss material to get impedance matching, while the multiple metal MOF-derived PC-based MAMs have shown excellent MA performance. They usually have multiple loss mechanism, so the synergistic effect between each part will be beneficial to impedance matching and electromagnetic wave attenuation. The structure of MOF also has a significant effect on the MA performance. Through design of MOF with different structures such as foam structure, core–shell structure, hollow structure, etc., the multiple reflections and interfacial polarization can be achieved. Therefore, MOF-derived PC-based nanocomposite is a promising material in the field of high-performance MAMs in the future.

**Table 2:** The MA performance of different kinds of MOF-derived PC-based nanocomposites as MAMs

Type	MAMs	*RL*_max_			EAB (< − 10 dB) (GHz)		Refs.
		Value (dB)	*f*_m_ (GHz)	Thickness (mm)	Value (GHz)	Thickness (mm)	
MOF-derived PC-based nanocomposites	Co/C-500	− 35.3	5.8	4	5.8 (8.4–14.2)	2.5	[123]
	Co/C-650	− 47.6	5.11	2	5.1 (12.1–17.2)	2	[124]
	Co/C-700	− 15.7	15.1	1.7	5.4 (12.3–17.7)	1.7	[125]
	Co/C-700	− 30.31	11.03	3	4.93 (8.31–13.24)	3	[126]
	Co/C-800	− 39.6	9.6	2	3.8 (10.7–14.5)	2	[127]
	MOF/RGO-500	− 52	9.6	4.1	7.72 (10.28–18)	3.2	[128]
	CoC-rGO-2	− 44.77	12.1	2.1	5.2 (12.8–18)	1.8	[129]
	Co_3_O_4_/Co/RGO	− 52.8	13.12	2	10.72 (4.88–15.6)	2–4	[130]
	3D Co/C-MCNT	− 20.3	13.84	1.8	4.08	1.8	[131]
	Co/C-MCNTs	− 48.9	9	2.99	–	–	[132]
	Co/CNT	− 49.16	14.16	2.5	4.2 (12.4–16.6)	2.5	[133]
	MWCNTs@carbonaceous CoO	− 50.2	14.3	1.84	4.32 (12.32–16.64)	1.84	[134]
	CNFs@carbonaceous Co/CoO	− 53.1	6.56	3.54	13.52 (3.68–14.64, 15.44–18)	2–5	[135]
	Co/C@PPy	− 44.76	17.32	2.0	6.56 (11.04–17.60)	2.5	[136]
	Co/NPC@ZnO/rGO	− 45.4	14.2	2	5.4 (11.9–17.3)	2	[138]
	Co/C@V_2_O_3_	− 40.1	14.1	1.5	4.64 (13.36–18)	1.5	[139]
	MnO@Co/C	−49.06	6.48	3.4	2.24	3.4	[140]
	Co/N/C@MnO_2_	− 58.9	5.56	3.7	5.5	–	[141]
	NRGO/MWCNT	−69.6	12.5	1.8	4.3 (13.2 − 17.5)	1.5	[143]
	Co/CNTs/CS	−51.2	12	2.2	4.1 (10.3 − 14.4)	2.2	[144]
	Co/C-HS	− 66.5	17.6	1.53	14.3 (3.7–18.0)	1–5	[145]
Ni-MOF-derived PC-based nanocomposites	Ni@C-800	− 57	13.8	1.85	6 (12–18)	1–5	[146]
	NPC/Ni	− 39.4	–	–	4.2	–	[147]
	Ni@C-ZIF	− 86.8	7.25	2.7	7.4 (4–11.4)	1.5–4	[149]
	SiC/Ni/NiO/C	− 50.52	13	4	2.96 (14.76–17.72)	2.5	[150]
	NiO/Ni/GN@Air@NiO/Ni/GN	− 34.5	17.2	1.7	10.2 (7.8–18)	1.7–5.0	[151]
	Porous flower-like Ni/C	− 52.4	16.1	1.6	5	1.6	[152]
Fe-MOF-derived PC-based nanocomposites	Fe/C nanocubes	− 22.6	4	5	7.2 (10.8–18)	2	[153]
	FC-650	− 39.43	14	2	5.36 (11.76–17.12)	2	[154]
	Fe/C-700-101	− 59.2	5	4.32	5	1.8	[155]
	MOF (Fe)/PANI	− 41.4	11.6	2	5.5 (9.8–15.3)	2	[156]
	Fe_3_O_4_@NPC	− 65.5	9.8	3	4.5	3	[157]
Zn-MOF-derived PC-based nanocomposites	ZnO/NPC/RGO	− 50.5	14	2.4	7.4 (9.6–17)	2.6	[158]
	Fe_3_O_4_/CNT	− 43	15.2	1.5	8.3 (9.7–18)	1.75	[159]
	PPy/ZIFs	− 49	12.1	2.9	7.24 (10.76–18)	2.6	[160]
Ti-MOF-derived PC-based nanocomposites	TiO_2_/C	− 49.6	15.8	1.6	4.6 (13.4–18 GHz)	1.6	[161]
Cu-MOF-derived PC-based nanocomposites	Ni/NiO/Cu@C	− 38.1	14.8	3.2	–	–	[162]
Zr-MOF-derived PC-based nanocomposites	ZrO_2_/C	− 57.2	15.8	3.3	11.9 (6.1–18)	3.3	[44]
	ZrO_2_/C	− 58.7	16.8	1.5	14.6 (3.4–18.0)	1–5	[163]
RE-MOF-derived PC-based nanocomposites	[Yb_2_(MH)_6_]_n_	− 28.14	–	2	–	–	[13]
	[Y_2_(MH)_6_]_n_·DMF	–	–	–	2.24 (6.8–9.04)	5	
Multi-magnetic metal MOF-derived PC-based nanocomposites	NiCo/NPC	− 51	17.9	1.5	4.5 (13.5–18)	1.5	[164]
	CoNi@C	− 44.8	6.8	3.2	13.3 (4.7–18.0)	1.6–4	[165]
	CoNi/C	− 74.7	15.6	1.8	15.1 (2.9–18.0)	0.3–5	[166]
	FeCo alloy/carbon	− 57.4	17.7	1.26	4.2 (11.0–15.2)	–	[167]
	Fe_3_Ni/C	− 46.2	10.44	2.65	5.24 (12.76–18)	2	[168]
	FeCoNi@C	− 69.03	5.52	2.1	8.08 (9.92–18)	2.47	[169]
	Core–shell FeCo@carbon/PDA	− 67.8	15.8	1.75	5.3 (11.0–16.3)	2	[170]
	NiCo alloy/C nanorod@CNT	− 58.8	14.0	2.2	6.5 (11.5–18)	2.2	[171]
	Fe–Co/NC/rGO	− 43.26	11.28	2.5	9.12 (8.88–18)	2.63	[172]
	FeNi@CNT/CNRs	− 47.0	–	2.3	4.5	1.6	[173]
	CoFe@C@MnO_2_ nanocubes	− 64	15.6	1.3	9.2 (8.8–18)	1.6	[174]
Magnetic and non-magnetic metal MOF-derived PC-based nanocomposites	CoO/Co/C	− 66.7	7.2	3.3	5.1 (12.6–17.7)	1.8	[175]
	Co@pNGC	− 50.7	11.3	2.5	4.0 (12.2–16.2)	1.2	[176]
Comparison MAMs	CNT	− 21	5	3.5	0.5	3.5	[177]
	rGO	− 6.9	7	2	–	–	[178]
	graphene foam	− 34	13.1	–	14.3 (3.7–18)	–	[59]
	Carbon nanotube/graphene foams	− 39.5	11.6	–	16	–	[179]
	Carbon nanotube grown on the carbon fiber	− 42	11.4	2.5	2.7	2.5	[180]
	3D PPy aerogel	− 22.5	12	3	5.0 (10.0–15.0)	3	[181]
	PANI nanoparticle	− 18.8	17.2	2	3.9 (14.1–18.0)	2	[182]
	ZnO nanoparticles	− 37.7	8.96	2.1	3.55 (7.5–11.05)	2.1	[183]
	C_3_N_4_ nanosheets	− 36.1	14.6	19.5	1.7	19.5	[184]
	SiC	− 24.8	11	3	4.2 (8.2–12.4)	3	[185]
	Fe powder	− 5.2	11	3	–	–	[186]
	Fe_3_O_4_@C	− 40	15.9	1.5	3.9 (14.1–18)	1.5	[93]
	Flaky carbonyl iron particles	− 14	0.6	1	1.6 (0.4–2)	1	[187]

## Conclusion

The recent progress of MOF-derived PC-based nanocomposites as MAMs has been systematically summarized by this review. In view of these studies, we find that MOF-derived PC-based MAMs from in situ pyrolysis of MOFs will be a promising method for the development of lightweight and highly effective MAMs. After pyrolysis, the PC-based MAMs from the MOFs exhibit porosity, low density, good electrical conductivity and dielectric loss. And the inorganic metal center can result in magnetic loss (magnetic metal) or dielectric loss (non-magnetic metal). To further improve the MA performance, the MOF-derived PC-based nanocomposites often coupled with other loss material. The well-designed nanocomposites with multiple advantages will show good impedance matching and strong EM attenuation capability because of the multiple loss mechanism and synergistic effect of the multi-components. Therefore, the MOF-derived PC-based nanocomposites coupled with multiple loss material are an attractive development direction of MAMs in the future.

Many achievements have been made in MOF-derived PC-based nanocomposites as MAMs, but most are just at the research stage, far away from the practical use. The *EAB* and the maximum *RL* values are not enough to meet the actual needs. We can rationally design the MAMs with the suitable preparation conditions to realize the special microstructure, which can improve the scattering of EM, and the multiple loss mechanism is realized by the synergistic effect of multiple components. As more than 20,000 kinds of MOFs have been used in various fields, we only review the common Ni, Co, Fe, Zn, Ti, Cu, Zr and RE metal as the central elements, and they have shown considerable MA performance. But they are just the tip of the iceberg of the big family of MOFs. We should also pay more attention to other metal elements. Through the modulation of inorganic metal center and organic ligand, different kinds of MOFs are constructed to achieve the optimal MA performance. The “thin, wide, light, strong” is the goal to develop MAMs, but most of the studies merely focus on the EAB and the maximum *RL* values, while the thickness and the weight of the MAMs have been usually ignored. In fact, low density is also an important parameter to evaluate the MAMs. One of the pyrolysis products of MOFs is carbon; therefore, MOFs are promising materials to employ new lightweight MAMs, especially in military applications. In conclusion, MOF-derived PC-based nanocomposites had already shown its great potential as MAMs. We firmly believe that the MOF-derived PC-based nanocomposites will be widely used in the field of MAMs in the future.

## References

[1] Wu N, Xu D, Wang Z, Wang F, Liu J (2019). Achieving superior electromagnetic wave absorbers through the novel metal-organic frameworks derived magnetic porous carbon nanorods. Carbon.

[2] Lv H, Liang X, Ji G, Zhang H, Du Y (2015). Porous three-dimensional flower-like Co/CoO and its excellent electromagnetic absorption properties. ACS Appl. Mater. Interfaces..

[3] Xie S, Ji Z, Zhu L, Zhang J, Cao Y (2020). Recent progress in electromagnetic wave absorption building materials. J. Build. Eng..

[4] Heng L, Zhang Z, Chen X, Wang S, Wu Z (2019). Fe/nanoporous carbon hybrid derived from metal-organic framework for highly effective microwave absorption. Appl. Organomet. Chem..

[5] Rehman SU, Wang J, Luo Q, Sun M, Jiang L (2019). Starfish-like C/CoNiO_2_ heterostructure derived from ZIF-67 with tunable microwave absorption properties. Chem. Eng. J..

[6] Qiao M, Lei X, Ma Y, Tian L, He X (2018). Application of yolk–shell Fe_3_O_4_@N-doped carbon nanochains as highly effective microwave-absorption material. Nano Res..

[7] Shen B, Zhai W, Tao M, Ling J, Zheng W (2013). Lightweight, multifunctional polyetherimide/graphene@Fe_3_O_4_ composite foams for shielding of electromagnetic pollution. ACS Appl. Mater. Interfaces..

[8] Liu JL, Liang HS, Zhang Y, Wu GL, Wu HJ (2019). Facile synthesis of ellipsoid-like MgCo_2_O_4_/Co_3_O_4_ composites for strong wideband microwave absorption application. Compos. Part B Eng..

[9] Zhou XF, Jia ZR, Feng AL, Wang XX, Liu JJ (2019). Synthesis of fish skin-derived 3D carbon foams with broadened bandwidth and excellent electromagnetic wave absorption performance. Carbon.

[10] Liu P, Huang Y, Yan J, Yang Y, Zhao Y (2016). Construction of CuS nanoflakes vertically aligned on magnetically decorated graphene and their enhanced microwave absorption properties. ACS Appl. Mater. Interfaces..

[11] Lu M-M, Cao M-S, Chen Y-H, Cao W-Q, Liu J (2015). Multiscale assembly of grape-like ferroferric oxide and carbon nanotubes: a smart absorber prototype varying temperature to tune intensities. ACS Appl. Mater. Interfaces..

[12] Ding D, Wang Y, Li X, Qiang R, Xu P (2017). Rational design of core-shell Co@C microspheres for high-performance microwave absorption. Carbon.

[13] Zhu LW, Liu N, Jiang XH, Yu LM, Li X (2020). Four novel 3D RE-MOFs based on maleic hydrazide: syntheses, structural diversity, efficient electromagnetic wave absorption and antibacterial activity properties. Inorg. Chim. Acta.

[14] Zhang Y, Wang X, Cao M (2018). Confinedly implanted NiFe_2_O_4_-rGO: cluster tailoring and highly tunable electromagnetic properties for selective-frequency microwave absorption. Nano Res..

[15] Saini P, Arora M, Gupta G, Gupta BK, Singh VN (2013). High permittivity polyaniline-barium titanate nanocomposites with excellent electromagnetic interference shielding response. Nanoscale.

[16] Huang L, Li J, Wang Z, Li Y, He X (2019). Microwave absorption enhancement of porous C@CoFe_2_O_4_ nanocomposites derived from eggshell membrane. Carbon.

[17] Xu Z, Du Y, Liu D, Wang Y, Ma W (2019). Pea-like Fe/Fe_3_C nanoparticles embedded in nitrogen-doped carbon nanotubes with tunable dielectric/magnetic loss and efficient electromagnetic absorption. ACS Appl. Mater. Interfaces..

[18] Wang X-X, Ma T, Shu J-C, Cao M-S (2018). Confinedly tailoring Fe_3_O_4_ clusters-NG to tune electromagnetic parameters and microwave absorption with broadened bandwidth. Chem. Eng. J..

[19] Jia Z, Lan D, Lin K, Qin M, Kou K (2018). Progress in low-frequency microwave absorbing materials. J. Mater. Sci.: Mater. Electron..

[20] Zhao H, Cheng Y, Liu W, Yang L, Zhang B (2019). Biomass-derived porous carbon-based nanostructures for microwave absorption. Nano-Micro Lett..

[21] Zhao H, Cheng Y, Lv H, Ji G, Du Y (2019). A novel hierarchically porous magnetic carbon derived from biomass for strong lightweight microwave absorption. Carbon.

[22] Ghosh S, Remanan S, Mondal S, Ganguly S, Das P (2018). An approach to prepare mechanically robust full IPN strengthened conductive cotton fabric for high strain tolerant electromagnetic interference shielding. Chem. Eng. J..

[23] Srogi K (2007). Microwave-assisted sample preparation of coal and coal fly ash for subsequent metal determination. Anal. Lett..

[24] Zhang X, Qiao J, Wang F, Lv L, Xu D (2020). Tailoring electromagnetic absorption performances of TiO_2_/Co/carbon nanofibers through tuning graphitization degrees. Ceram. Int..

[25] Zhao B, Shao G, Fan B, Zhao W, Xie Y (2015). Synthesis of flower-like CuS hollow microspheres based on nanoflakes self-assembly and their microwave absorption properties. J. Mater. Chem. A.

[26] Munir A (2017). Microwave radar absorbing properties of multiwalled carbon nanotubes polymer composites: a review. Adv. Polym. Tech..

[27] Safaei M, Foroughi MM, Ebrahimpoor N, Jahani S, Omidi A (2019). A review on metal-organic frameworks: synthesis and applications. Trac-Trends Anal. Chem..

[28] Xu X, Ran F, Fan Z, Lai H, Cheng Z (2019). Cactus-inspired bimetallic metal-organic framework-derived 1D-2D hierarchical Co/N-decorated carbon architecture toward enhanced electromagnetic wave absorbing performance. ACS Appl. Mater. Interfaces..

[29] Wang Y, Gao X, Lin C, Shi L, Li X (2019). Metal organic frameworks-derived Fe-Co nanoporous carbon/graphene composite as a high-performance electromagnetic wave absorber. J. Alloys Compd..

[30] Ye Y, Ma Z, Lin R-B, Krishna R, Zhou W (2019). Pore space partition within a metal-organic framework for highly efficient C_2_H_2_/CO_2_ separation. J. Am. Chem. Soc..

[31] Zhang H, Liu X, Wu Y, Guan C, Cheetham AK (2018). MOF-derived nanohybrids for electrocatalysis and energy storage: current status and perspectives. Chem. Commun..

[32] Cai Z-X, Wang Z-L, Kim J, Yamauchi Y (2019). Hollow functional materials derived from metal–organic frameworks: synthetic strategies, conversion mechanisms, and electrochemical applications. Adv. Mater..

[33] Liang Z, Qu C, Guo W, Zou R, Xu Q (2018). Pristine metal-organic frameworks and their composites for energy storage and conversion. Adv. Mater..

[34] Wei X, Li Y, Peng H, Zhou M, Ou Y (2018). Metal-organic framework-derived hollow CoS nanobox for high performance electrochemical energy storage. Chem. Eng. J..

[35] Li J, Huang W, Wang M, Xi S, Meng J (2019). Low-crystalline bimetallic metal-organic framework electrocatalysts with rich active sites for oxygen evolution. ACS Energy Lett..

[36] Zhu B, Zou R, Xu Q (2018). Metal-organic framework based catalysts for hydrogen evolution. Adv. Energy Mater..

[37] Lin R-B, Xiang S, Xing H, Zhou W, Chen B (2019). Exploration of porous metal-organic frameworks for gas separation and purification. Coord. Chem. Rev..

[38] Jiang X, Li S, Bai Y, Shao L (2019). Ultra-facile aqueous synthesis of nanoporous zeolitic imidazolate framework membranes for hydrogen purification and olefin/paraffin separation. J. Mater. Chem. A.

[39] Chang J, Wang X, Wang J, Li H, Li F (2019). Nucleic acid-functionalized metal-organic framework-based homogeneous electrochemical biosensor for simultaneous detection of multiple tumor biomarkers. Anal. Chem..

[40] Li Y, Xie M, Zhang X, Liu Q, Lin D, Xu C, Xie F, Sun X (2019). Co-MOF nanosheet array: a high-performance electrochemical sensor for non-enzymatic glucose detection. Sens. Actuat. B Chem..

[41] Liu W, Tan S, Yang Z, Ji G (2018). Enhanced low-frequency electromagnetic properties of MOF-derived cobalt through interface design. ACS Appl. Mater. Interfaces..

[42] Xu H, Yin X, Zhu M, Li M, Zhang H (2019). Constructing hollow graphene nano-spheres confined in porous amorphous carbon particles for achieving full X band microwave absorption. Carbon.

[43] Zhang Y, Gao S, Xing H, Li H (2019). In situ carbon nanotubes encapsulated metal Nickel as high-performance microwave absorber from Ni-Zn metal-organic framework derivative. J. Alloys Compd..

[44] Zhang X, Qao J, Zhao J, Xu D, Wang F (2019). High-efficiency electromagnetic wave absorption of cobalt decorated NH_2_-UIO-66-derived porous ZrO_2_/C. ACS Appl. Mater. Interfaces..

[45] Yang W, Li X, Li Y, Zhu R, Pang H (2019). Applications of metal-organic-framework-derived carbon materials. Adv. Mater..

[46] Liu W, Liu L, Yang Z, Xu J, Hou Y (2018). A versatile route toward the electromagnetic functionalization of metal–organic framework-derived three-dimensional nanoporous carbon composites. ACS Appl. Mater. Interfaces..

[47] Wu Q, Jin H, Chen W, Huo S, Chen X (2018). Graphitized nitrogen-doped porous carbon composites derived from ZIF-8 as efficient microwave absorption materials. Mater. Res. Express.

[48] Meng F, Wang H, Huang F, Guo Y, Wang Z (2018). Graphene-based microwave absorbing composites: a review and prospective. Compos. Part B Eng..

[49] Shu R, Zhang J, Guo C, Wu Y, Wan Z (2020). Facile synthesis of nitrogen-doped reduced graphene oxide/nickel-zinc ferrite composites as high-performance microwave absorbers in the X-band. Chem. Eng. J..

[50] Wu Y, Shu R, Shan X, Zhang J, Shi J (2020). Facile design of cubic-like cerium oxide nanoparticles decorated reduced graphene oxide with enhanced microwave absorption properties. J. Alloys Compd..

[51] Yan J, Huang Y, Chen C, Liu X, Liu H (2019). The 3D CoNi alloy particles embedded in N-doped porous carbon foams for high-performance microwave absorbers. Carbon.

[52] Wu Z, Tian K, Huang T, Hu W, Xie F (2018). Hierarchically porous carbons derived from biomasses with excellent microwave absorption performance. ACS Appl. Mater. Interfaces..

[53] Wu N, Liu C, Xu D, Liu J, Liu W (2018). Enhanced electromagnetic wave absorption of three-dimensional porous Fe_3_O_4_/C composite flowers. ACS Sustain. Chem. Eng..

[54] Zeng M, Cao Q, Liu J, Guo B, Hao X (2020). Hierarchical cobalt selenides as highly efficient microwave absorbers with tunable frequency response. ACS Appl. Mater. Interfaces..

[55] Yang LJ, Lv HL, Li M, Zhang Y, Liu JC (2020). Multiple polarization effect of shell evolution on hierarchical hollow C@MnO_2_ composites and their wideband electromagnetic wave absorption properties. Chem. Eng. J..

[56] Wang Z, Wei R, Gu J, Liu H, Liu C (2018). Ultralight, highly compressible and fire-retardant graphene aerogel with self-adjustable electromagnetic wave absorption. Carbon.

[57] Wang K, Chen Y, Tian R, Li H, Zhou Y (2018). Porous Co-C core-shell nanocomposites derived from Co-MOF-74 with enhanced electromagnetic wave absorption performance. ACS Appl. Mater. Interfaces..

[58] Shu R, Zhang G, Wang X, Gao X, Wang M (2018). Fabrication of 3D net-like MWCNTs/ZnFe_2_O_4_ hybrid composites as high-performance electromagnetic wave absorbers. Chem. Eng. J..

[59] Zhang Y, Huang Y, Chen H, Huang Z, Yang Y (2016). Composition and structure control of ultralight graphene foam for high-performance microwave absorption. Carbon.

[60] Zhang J, Shu R, Wu Y, Wan Z, Zheng M (2019). Facile fabrication and enhanced microwave absorption properties of reduced graphene oxide/tin dioxide binary nanocomposites in the X-band. Synth. Met..

[61] Song Q, Ye F, Kong L, Shen Q, Han L (2020). Graphene and MXene nanomaterials: toward high-performance electromagnetic wave absorption in gigahertz band range. Adv. Funct. Mater..

[62] Shao G, Liang J, Zhao W, Zhao B, Liu W (2020). Co decorated polymer-derived SiCN ceramic aerogel composites with ultrabroad microwave absorption performance. J. Alloys Compd..

[63] Dong S, Hu P, Li X, Hong C, Zhang X (2020). NiCo_2_S_4_ nanosheets on 3D wood-derived carbon for microwave absorption. Chem. Eng. J..

[64] Wang X, Pan F, Xiang Z, Zeng Q, Pei K (2020). Magnetic vortex core-shell Fe_3_O_4_@C nanorings with enhanced microwave absorption performance. Carbon.

[65] Dong S, Zhang W, Zhang X, Hu P, Han J (2018). Designable synthesis of core-shell SiCw@C heterostructures with thickness-dependent electromagnetic wave absorption between the whole X-band and Ku-band. Chem. Eng. J..

[66] Liu L, Yang S, Hu H, Zhang T, Yuan Y (2019). Lightweight and efficient microwave-absorbing materials based on loofah-sponge-derived hierarchically porous carbons. ACS Sustain. Chem. Eng..

[67] Wang H, Meng F, Huang F, Jing C, Li Y (2019). Interface modulating CNTs@PANi hybrids by controlled unzipping of the walls of CNTs to achieve tunable high-performance microwave absorption. ACS Appl. Mater. Interfaces..

[68] Kuang J, Jiang P, Ran F, Cao W (2016). Conductivity-dependent dielectric properties and microwave absorption of Al-doped SiC whiskers. J. Alloys Compd..

[69] Qi X, Yang Y, Zhong W, Qin C, Deng Y (2010). Simultaneous synthesis of carbon nanobelts and carbon/Fe–Cu hybrids for microwave absorption. Carbon.

[70] Xu H, Yin X, Li X, Li M, Liang S (2019). Lightweight Ti_2_CT_x_ MXene/Poly(vinyl alcohol) composite foams for electromagnetic wave shielding with absorption-dominated feature. ACS Appl. Mater. Interfaces..

[71] Jayalakshmi CG, Inamdar A, Anand A, Kandasubramanian B (2019). Polymer matrix composites as broadband radar absorbing structures for stealth aircrafts. J. Appl. Polym. Sci..

[72] Jiang J, Li D, Geng D, An J, He J (2014). Microwave absorption properties of core double-shell FeCo/C/BaTiO_3_ nanocomposites. Nanoscale.

[73] Liu P, Huang Y, Yan J, Zhao Y (2016). Magnetic graphene@PANI@porous TiO_2_ ternary composites for high-performance electromagnetic wave absorption. J. Mater. Chem. C.

[74] Duan W, Yin X, Li Q, Schlier L, Greil P (2016). A review of absorption properties in silicon-based polymer derived ceramics. J. Eur. Ceram. Soc..

[75] Zhou W, Yin R-M, Long L, Luo H, Hu W-D (2018). Enhanced high-temperature dielectric properties and microwave absorption of SiC nanofibers modified Si_3_N_4_ ceramics within the gigahertz range. Ceram. Int..

[76] Lv H, Liang X, Cheng Y, Zhang H, Tang D (2015). Coin-like alpha-Fe_2_O_3_@CoFe_2_O_4_ core-shell composites with excellent electromagnetic absorption performance. ACS Appl. Mater. Interfaces..

[77] Quan B, Lang X, Ji G, Ma J, Ouyang P (2017). Strong electromagnetic wave response derived from the construction of dielectric/magnetic media heterostructure and multiple interfaces. ACS Appl. Mater. Interfaces..

[78] Zhou C, Wang X, Luo H, Deng L, Wang S (2019). Interfacial design of sandwich-like CoFe@Ti_3_C_2_T_x_ composites as high efficient microwave absorption materials. Appl. Surf. Sci..

[79] Li N, Xie X, Lu H, Fan B, Wang X (2019). Novel two-dimensional Ti_3_C_2_T_x_/Ni-spheres hybrids with enhanced microwave absorption properties. Ceram. Int..

[80] Lou Z, Yuan C, Zhang Y, Li Y, Cai J (2019). Synthesis of porous carbon matrix with inlaid Fe_3_C/Fe_3_O_4_ micro-particles as an effective electromagnetic wave absorber from natural wood shavings. J. Alloys Compd..

[81] Wu T, Liu Y, Zeng X, Cui T, Zhao Y (2016). Facile hydrothermal synthesis of fe_3_o_4_/c core-shell nanorings for efficient low-frequency microwave absorption. ACS Appl. Mater. Interfaces..

[82] Almessiere MA, Slimani Y, Güngüneş H, Kostishyn VG, Trukhanov SV (2020). Impact of Eu^3+^ ion substitution on structural, magnetic and microwave traits of Ni–Cu–Zn spinel ferrites. Ceram. Int..

[83] Klygach DS, Vakhitov MG, Vinnik DA, Bezborodov AV, Gudkova SA (2018). Measurement of permittivity and permeability of barium hexaferrite. J. Magn. Magn. Mater..

[84] Vinnik DA, Klygach DS, Zhivulin VE, Malkin AI, Vakhitov MG (2018). Electromagnetic properties of BaFe12O19: Ti at centimeter wavelengths. J. Alloys Compd..

[85] Trukhanov AV, Almessiere MA, Baykal A, Trukhanov SV, Slimani Y (2019). Influence of the charge ordering and quantum effects in heterovalent substituted hexaferrites on their microwave characteristics. J. Alloys Compd..

[86] Matzui LY, Trukhanov AV, Yakovenko OS, Vovchenko LL, Zagorodnii VV (2019). Functional magnetic composites based on hexaferrites: correlation of the composition. Magnetic and high-frequency properties. Nanomaterials.

[87] Yakovenko OS, Matzui LY, Vovchenko LL, Trukhanov AV, Kazakevich IS (2017). Magnetic anisotropy of the graphite nanoplatelet–epoxy and MWCNT–epoxy composites with aligned barium ferrite filler. J. Mater. Sci..

[88] Qing Y, Min D, Zhou Y, Luo F, Zhou W (2015). Graphene nanosheet- and flake carbonyl iron particle-filled epoxy-silicone composites as thin-thickness and wide-bandwidth microwave absorber. Carbon.

[89] Khani O, Shoushtari MZ, Ackland K, Stamenov P (2017). The structural, magnetic and microwave properties of spherical and flake shaped carbonyl iron particles as thin multilayer microwave absorbers. J. Magn. Magn. Mater..

[90] Yan S, Cao C, He J, He L, Qu Z (2019). Investigation on the electromagnetic and broadband microwave absorption properties of Ti_3_C_2_ Mxene/flaky carbonyl iron composites. J. Mater. Sci.: Mater. Electron..

[91] Li Q, Zhang Z, Qi L, Liao Q, Kang Z (2019). Toward the application of high frequency electromagnetic wave absorption by carbon nanostructures. Adv. Sci..

[92] Fu C, He D, Wang Y, Zhao X (2019). Enhanced microwave absorption performance of RGO-modified Co@C nanorods. Synth. Met..

[93] Du Y, Liu W, Qiang R, Wang Y, Han X (2014). Shell thickness-dependent microwave absorption of core-shell Fe_3_O_4_@C composites. ACS Appl. Mater. Interfaces..

[94] Zhang Y, Huang Y, Zhang T, Chang H, Xiao P (2015). Broadband and tunable high-performance microwave absorption of an ultralight and highly compressible graphene foam. Adv. Mater..

[95] Zhao H-B, Cheng J-B, Zhu J-Y, Wang Y-Z (2019). Ultralight CoNi/rGO aerogels toward excellent microwave absorption at ultrathin thickness. J. Mater. Chem. C.

[96] Zhang H, Wang B, Feng A, Zhang N, Jia Z (2019). Mesoporous carbon hollow microspheres with tunable pore size and shell thickness as efficient electromagnetic wave absorbers. Compos. Part B Eng..

[97] Wen B, Cao M-S, Hou Z-L, Song W-L, Zhang L (2013). Temperature dependent microwave attenuation behavior for carbon-nanotube/silica composites. Carbon.

[98] Quan B, Shi W, Ong SJH, Lu X, Wang PL (2019). Defect engineering in two common types of dielectric materials for electromagnetic absorption applications.

[99] Lv H, Zhang H, Ji G, Xu ZJ (2016). Interface strategy to achieve tunable high frequency attenuation. ACS Appl. Mater. Interfaces..

[100] Sun G, Dong B, Cao M, Wei B, Hu C (2011). Hierarchical dendrite-like magnetic materials of Fe_3_O_4_, gamma-Fe_2_O_3_, and Fe with high performance of microwave absorption. Chem. Mater..

[101] Cao M-S, Wang X-X, Zhang M, Shu J-C, Cao W-Q (2019). Electromagnetic response and energy conversion for functions and devices in low-dimensional materials. Adv. Funct. Mater..

[102] Liang X, Quan B, Man Z, Cao B, Li N (2019). Self-assembly three-dimensional porous carbon networks for efficient dielectric attenuation. ACS Appl. Mater. Interfaces..

[103] Liu Q, Liu X, Feng H, Shui H, Yu R (2017). Metal organic framework-derived Fe/carbon porous composite with low Fe content for lightweight and highly efficient electromagnetic wave absorber. Chem. Eng. J..

[104] Li N, Huang G-W, Li Y-Q, Xiao H-M, Feng Q-P (2017). Enhanced microwave absorption performance of coated carbon nanotubes by optimizing the Fe_3_O_4_ nanocoating structure. ACS Appl. Mater. Interfaces..

[105] Cheng Y, Seow JZY, Zhao H, Xu ZJ, Ji G (2020). A flexible and lightweight biomass-reinforced microwave absorber. Nano-Micro Lett..

[106] Yang Y, Xia L, Zhang T, Shi B, Huang L (2018). Fe_3_O_4_@LAS/RGO composites with a multiple transmission-absorption mechanism and enhanced electromagnetic wave absorption performance. Chem. Eng. J..

[107] Zhang N, Huang Y, Zong M, Ding X, Li S (2017). Synthesis of ZnS quantum dots and CoFe_2_O_4_ nanoparticles co-loaded with graphene nanosheets as an efficient broad band EM wave absorber. Chem. Eng. J..

[108] Lv H, Guo Y, Wu G, Ji G, Zhao Y (2017). Interface polarization strategy to solve electromagnetic wave interference issue. ACS Appl. Mater. Interfaces..

[109] Lin Y, Dai J, Yang H, Wang L, Wang F (2018). Graphene multilayered sheets assembled by porous Bi_2_Fe_4_O_9_ microspheres and the excellent electromagnetic wave absorption properties. Chem. Eng. J..

[110] Hanai T (1960). Theory of the dielectric dispersion due to the interfacial polarization and its application to emulsions. Kolloid-Zeitschrift.

[111] Goodenough JB (2002). Summary of losses in magnetic materials. IEEE Trans. Magn..

[112] Cao M-S, Shu J-C, Wang X-X, Wang X, Zhang M (2019). Electronic structure and electromagnetic properties for 2D electromagnetic functional materials in gigahertz frequency. Ann. Phys..

[113] Zhao B, Zhao W, Shao G, Fan B, Zhang R (2015). Morphology-control synthesis of a core-shell structured NiCu alloy with tunable electromagnetic-wave absorption capabilities. ACS Appl. Mater. Interfaces..

[114] Carrey J, Mehdaoui B, Respaud M (2011). Simple models for dynamic hysteresis loop calculations of magnetic single-domain nanoparticles: application to magnetic hyperthermia optimization. J. Appl. Phys..

[115] Zhu C-L, Zhang M-L, Qiao Y-J, Xiao G, Zhang F (2010). Fe_3_O_4_/TiO_2_ core/shell nanotubes: synthesis and magnetic and electromagnetic wave absorption characteristics. J. Phys. Chem. C.

[116] Xu D, Xiong X, Chen P, Yu Q, Chu H (2019). Superior corrosion-resistant 3D porous magnetic graphene foam-ferrite nanocomposite with tunable electromagnetic wave absorption properties. J. Magn. Magn. Mater..

[117] Liang X, Man Z, Quan B, Zheng J, Gu W (2020). Environment-stable Co_x_Ni_y_ encapsulation in stacked porous carbon nanosheets for enhanced microwave absorption. Nano-Micro Lett..

[118] Shu R, Wu Y, Li Z, Zhang J, Wan Z (2019). Facile synthesis of cobalt-zinc ferrite microspheres decorated nitrogen-doped multi-walled carbon nanotubes hybrid composites with excellent microwave absorption in the X-band. Compos. Sci. Technol..

[119] Li Z, Li X, Zong Y, Tan G, Sun Y (2017). Solvothermal synthesis of nitrogen-doped graphene decorated by superparamagnetic Fe_3_O_4_ nanoparticles and their applications as enhanced synergistic microwave absorbers. Carbon.

[120] Wu Y, Shu R, Zhang J, Wan Z, Shi J (2020). Oxygen vacancies regulated microwave absorption properties of reduced graphene oxide/multi-walled carbon nanotubes/cerium oxide ternary nanocomposite. J. Alloys Compd..

[121] Zhao X, Wang Y, Li DS, Bu X, Feng P (2018). Metal-organic frameworks for separation. Adv. Mater..

[122] Zhao R, Liang Z, Zou R, Xu Q (2018). Metal-organic frameworks for batteries. Joule.

[123] Lu Y, Wang Y, Li H, Lin Y, Jiang Z (2015). MOF-derived porous Co/C nanocomposites with excellent electromagnetic wave absorption properties. ACS Appl. Mater. Interfaces..

[124] Li J, Miao P, Chen K-J, Cao J-W, Liang J (2020). Highly effective electromagnetic wave absorbing prismatic Co/C nanocomposites derived from cubic metal-organic framework. Compos. Part B Eng..

[125] Zhu B-Y, Miao P, Kong J, Zhang X-L, Wang G-Y (2019). Co/C composite derived from a newly constructed metal-organic framework for effective microwave absorption. Cryst. Growth Des..

[126] Wang H, Xiang L, Wei W, An J, He J (2017). Efficient and lightweight electromagnetic wave absorber derived from metal organic framework-encapsulated cobalt nanoparticles. ACS Appl. Mater. Interfaces..

[127] Qiang R, Du Y, Chen D, Ma W, Wang Y (2016). Electromagnetic functionalized Co/C composites by in situ pyrolysis of metal-organic frameworks (ZIF-67). J. Alloys Compd..

[128] Zhang K, Xie A, Sun M, Jiang W, Wu F (2017). Electromagnetic dissipation on the surface of metal organic framework (MOF)/reduced graphene oxide (RGO) hybrids. Mater. Chem. Phys..

[129] Qiu H, Zhu X, Chen P, Yang S, Guo X (2020). Magnetic dodecahedral CoC-decoratedreduced graphene oxide as excellent electromagnetic wave absorber. J. Electron. Mater..

[130] Yuan J, Liu Q, Li S, Lu Y, Jin S (2017). Metal organic framework (MOF)-derived carbonaceous Co_3_O_4_/Co microframes anchored on RGO with enhanced electromagnetic wave absorption performances. Synth. Met..

[131] Zhang K, Wu F, Li J, Sun M, Xie A (2018). Networks constructed by metal organic frameworks (MOFs) and multiwall carbon nanotubes (MCNTs) for excellent electromagnetic waves absorption. Mater. Chem. Phys..

[132] Yin Y, Liu X, Wei X, Li Y, Nie X (2017). Magnetically aligned Co-C/MWCNTs composite derived from MWCNT-interconnected zeolitic imidazolate frameworks for a lightweight and highly efficient electromagnetic wave absorber. ACS Appl. Mater. Interfaces..

[133] Xiao X, Zhu W, Tan Z, Tian W, Guo Y (2018). Ultra-small Co/CNTs nanohybrid from metal organic framework with highly efficient microwave absorption. Compos. Part B Eng..

[134] Lu S, Meng Y, Wang H, Wang F, Yuan J (2019). Great enhancement of electromagnetic wave absorption of MWCNTs@ carbonaceous CoO composites derived from MWCNTs-interconnected zeolitic imidazole framework. Appl. Surf. Sci..

[135] Chen H, Hong R, Liu Q, Li S, Huang F (2018). CNFs@carbonaceous Co/CoO composite derived from CNFs penetrated through ZIF-67 for high-efficient electromagnetic wave absorption material. J. Alloys Compd..

[136] Sun X, Lv X, Sui M, Weng X, Li X (2018). Decorating MOF-derived nanoporous Co/C in chain-like polypyrrole (PPy) aerogel: a lightweight material with excellent electromagnetic absorption. Materials.

[137] Liu X, Wang L-S, Ma Y, Qiu Y, Xie Q (2018). Facile synthesis and microwave absorption properties of yolk-shell ZnO-Ni-C/RGO composite materials. Chem. Eng. J..

[138] Kang S, Zhang W, Hu Z, Yu J, Wang Y (2020). Porous core-shell zeolitic imidazolate framework-derived Co/NPC@ZnO-decorated reduced graphene oxide for lightweight and broadband electromagnetic wave absorber. J. Alloys Compd..

[139] Zhou C, Wu C, Liu D, Yan M (2019). Metal-organic framework derived hierarchical Co/C@V_2_O_3_ hollow spheres as a thin, lightweight, and high-efficiency electromagnetic wave absorber. Chem. Eur. J..

[140] Liu M, Tian R, Chen H, Li S, Huang F (2020). One-dimensional chain-like MnO@Co/C composites for high-efficient electromagnetic wave absorbent. J. Magn. Magn. Mater..

[141] Wang R, He M, Zhou Y, Nie S, Wang Y (2020). Metal-organic frameworks self-templated cubic hollow Co/N/C@MnO_2_ composites for electromagnetic wave absorption. Carbon.

[142] Zhang K, Wu F, Xie A, Sun M, Dong W (2017). In situ stringing of metal organic frameworks by SiC nanowires for high-performance electromagnetic radiation elimination. ACS Appl. Mater. Interfaces..

[143] Shu R, Wan Z, Zhang J, Wu Y, Liu Y (2020). Facile design of three-dimensional nitrogen-doped reduced graphene oxide/multi-walled carbon nanotube composite foams as lightweight and highly efficient microwave absorbers. ACS Appl. Mater. Interfaces..

[144] Yang N, Luo Z-X, Zhu G-R, Chen S-C, Wang X-L (2019). Ultralight three-dimensional hierarchical cobalt nanocrystals/N-doped CNTs/carbon sponge composites with a hollow skeleton toward superior microwave absorption. ACS Appl. Mater. Interfaces..

[145] Li Z, Han X, Ma Y, Liu D, Wang Y (2018). MOFs-derived hollow Co/C microspheres with enhanced microwave absorption performance. ACS Sustain. Chem. Eng..

[146] Zhang Z, Zhu Q, Chen X, Wu Z, He Y (2019). Ni@C composites derived from Ni-based metal organic frameworks with a lightweight, ultrathin, broadband and highly efficient microwave absorbing properties. Appl. Phys. Express.

[147] Quan B, Xu G, Yi H, Yang Z, Xiang J (2018). Enhanced electromagnetic wave response of nickel nanoparticles encapsulated in nanoporous carbon. J. Alloys Compd..

[148] Yang Z, Zhang Y, Li M, Yang L, Liu J (2019). Surface architecture of Ni-based metal organic framework hollow spheres for adjustable microwave absorption. ACS Appl. Nano Mater..

[149] Yan J, Huang Y, Yan Y, Ding L, Liu P (2019). High-performance electromagnetic wave absorbers based on two kinds of nickel-based MOF-derived Ni@C microspheres. ACS Appl. Mater. Interfaces..

[150] Yang R, Yuan J, Yu C, Yan K, Fu Y (2020). Efficient electromagnetic wave absorption by SiC/Ni/NiO/C nanocomposites. J. Alloys Compd..

[151] Liang X, Quan B, Sun Y, Ji G, Zhang Y (2017). Multiple interfaces structure derived from metal-organic frameworks for excellent electromagnetic wave absorption. Part. Part. Syst. Char..

[152] Zhang Z, Lv Y, Chen X, Wu Z, He Y (2019). Porous flower-like Ni/C composites derived from MOFs toward high-performance electromagnetic wave absorption. J. Magn. Magn. Mater..

[153] Qiang R, Du Y, Zhao H, Wang Y, Tian C (2015). Metal organic framework-derived Fe/C nanocubes toward efficient microwave absorption. J. Mater. Chem. A.

[154] Peng S, Wang S, Hao G, Zhu C, Zhang Y (2019). Preparation of magnetic flower-like carbon-matrix composites with efficient electromagnetic wave absorption properties by carbonization of MIL-101(Fe). J. Magn. Magn. Mater..

[155] Miao P, Zhou R, Chen K, Liang J, Ban Q (2020). Tunable Electromagnetic Wave Absorption Of Supramolecular Isomer-Derived Nanocomposites With Different Morphology. Adv. Mater. Interfaces.

[156] Wang Y, Zhang W, Wu X, Luo C, Wang Q (2017). Conducting polymer coated metal-organic framework nanoparticles: facile synthesis and enhanced electromagnetic absorption properties. Synth. Met..

[157] Xiang Z, Song Y, Xiong J, Pan Z, Wang X (2019). Enhanced electromagnetic wave absorption of nanoporous Fe_3_O_4_ @ carbon composites derived from metal-organic frameworks. Carbon.

[158] Liang X, Quan B, Ji G, Liu W, Zhao H (2017). Tunable dielectric performance derived from the metal-organic framework/reduced graphene oxide hybrid with broadband absorption. ACS Sustain. Chem. Eng..

[159] Gu W, Lv J, Quan B, Liang X, Zhang B (2019). Achieving MOF-derived one-dimensional porous ZnO/C nanofiber with lightweight and enhanced microwave response by an electrospinning method. J. Alloys Compd..

[160] Jiao Y, Li J, Xie A, Wu F, Zhang K (2019). Confined polymerization strategy to construct polypyrrole/zeolitic imidazolate frameworks (PPy/ZIFs) nanocomposites for tunable electrical conductivity and excellent electromagnetic absorption. Compos. Sci. Technol..

[161] Ma J, Liu W, Liang X, Quan B, Cheng Y (2017). Nanoporous TiO_2_/C composites synthesized from directly pyrolysis of a Ti-based MOFs MIL-125(Ti) for efficient microwave absorption. J. Alloys Compd..

[162] Huang L, Chen C, Huang X, Ruan S, Zeng Y-J (2019). Enhanced electromagnetic absorbing performance of MOF-derived Ni/NiO/Cu@C composites. Compos. Part B Eng..

[163] Zhang X, Qiao J, Liu C, Wang F, Jiang Y (2020). A MOF-derived ZrO_2_/C nanocomposite for efficient electromagnetic wave absorption. Inorg. Chem. Front..

[164] Xiong J, Xiang Z, Zhao J, Yu L, Cui E (2019). Layered NiCo alloy nanoparticles/nanoporous carbon composites derived from bimetallic MOFs with enhanced electromagnetic wave absorption performance. Carbon.

[165] Liu Y, Chen Z, Xie W, Qiu F, Zhang Y (2019). Enhanced microwave absorption performance of porous and hollow CoNi@C microspheres with controlled component and morphology. J. Alloys Compd..

[166] Liu C, Qiao J, Zhang X, Xu D, Wu N (2019). Bimetallic MOF-derived porous CoNi/C nanocomposites with ultra-wide band microwave absorption properties. New J. Chem..

[167] Liu D, Qiang R, Du Y, Wang Y, Tian C (2018). Prussian blue analogues derived magnetic FeCo alloy/carbon composites with tunable chemical composition and enhanced microwave absorption. J. Colloid Interface Sci..

[168] Liu W, Tan S, Yang Z, Ji G (2018). Hollow graphite spheres embedded in porous amorphous carbon matrices as lightweight and low-frequency microwave absorbing material through modulating dielectric loss. Carbon.

[169] Ouyang J, He Z, Zhang Y, Yang H, Zhao Q (2019). Trimetallic FeCoNi@C nanocomposite hollow spheres derived from metal-organic frameworks with superior electromagnetic wave absorption ability. ACS Appl. Mater. Interfaces..

[170] Wang F, Wang N, Han X, Liu D, Wang Y (2019). Core-shell FeCo@carbon nanoparticles encapsulated in polydopamine-derived carbon nanocages for efficient microwave absorption. Carbon.

[171] Wang L, Wen B, Bai X, Liu C, Yang H (2019). NiCo alloy/carbon nanorods decorated with carbon nanotubes for microwave absorption. ACS Appl. Nano Mater..

[172] Wang S, Xu Y, Fu R, Zhu H, Jiao Q (2019). Rational construction of hierarchically porous Fe-Co/N-doped carbon/rGO composites for broadband microwave absorption. Nano-Micro Lett..

[173] Xu X, Ran F, Lai H, Cheng Z, Lv T (2019). In Situ confined bimetallic metal-organic framework derived nanostructure within 3D interconnected bamboo-like carbon nanotube networks for boosting electromagnetic wave absorbing performances. ACS Appl. Mater. Interfaces..

[174] Zhang Y, Yang Z, Li M, Yang L, Liu J (2020). Heterostructured CoFe@C@MnO_2_ nanocubes for efficient microwave absorption. Chem. Eng. J..

[175] Shu R, Li W, Wu Y, Zhang J, Zhang G (2019). Fabrication of nitrogen-doped cobalt oxide/cobalt/carbon nanocomposites derived from heterobimetallic zeolitic imidazolate frameworks with superior microwave absorption properties. Compos. Part B Engn..

[176] Wang S, Ke X, Zhong S, Lai Y, Qian D, Wang Y, Wang Q, Jiang W (2017). Bimetallic zeolitic imidazolate frameworks-derived porous carbon-based materials with efficient synergistic microwave absorption properties: the role of calcining temperature. RSC Adv..

[177] Qi X, Xu J, Hu Q, Deng Y, Xie R (2016). Metal-free carbon nanotubes: synthesis, and enhanced intrinsic microwave absorption properties. Sci. Rep..

[178] Wang C, Han X, Xu P, Zhang X, Du Y (2011). The electromagnetic property of chemically reduced graphene oxide and its application as microwave absorbing material. Appl. Phys. Lett..

[179] Chen H, Huang Z, Huang Y, Zhang Y, Ge Z (2017). Synergistically assembled MWCNT/graphene foam with highly efficient microwave absorption in both C and X bands. Carbon.

[180] Singh SK, Akhtar MJ, Kar KK (2018). Hierarchical carbon nanotube-coated carbon fiber: ultra lightweight, thin, and highly efficient microwave sbsorber. ACS Appl. Mater. Interfaces..

[181] Xie A, Wu F, Sun M, Dai X, Xu Z (2015). Self-assembled ultralight three-dimensional polypyrrole aerogel for effective electromagnetic absorption. Appl. Phys. Lett..

[182] Zhang P, Han X, Kang L, Qiang R, Liu W (2013). Synthesis and characterization of polyaniline nanoparticles with enhanced microwave absorption. RSC Adv..

[183] Cai M, Shui A, Wang X, He C, Qian J (2020). A facile fabrication and high-performance electromagnetic microwave absorption of ZnO nanoparticles. J. Alloys Compd..

[184] Green M, Liu Z, Smedley R, Nawaz H, Li X (2018). Graphitic carbon nitride nanosheets for microwave absorption. Mater. Today Phys..

[185] Liang C, Wang Z, Wu L, Zhang X, Wang H (2017). Light and strong hierarchical porous SiC foam for efficient electromagnetic interference shielding and thermal insulation at elevated temperatures. ACS Appl. Mater. Interfaces..

[186] Yang RB, Liang WF, Lou CW, Lin JH (2012). Electromagnetic and microwave absorption properties of magnetic stainless steel powder in 2–18 GHz. J. Appl. Phys..

[187] Wang F, Long C, Wu T, Li W, Chen Z (2020). Enhancement of low-frequency magnetic permeability and absorption by texturing flaky carbonyl iron particles. J. Alloys Compd..

